# Supplementary material for: Treating common mental disorder including psychotic experiences in the primary care improving access to psychological therapies programme (the TYPPEX study): protocol for a stepped wedge cluster randomised controlled trial with nested economic and process evaluation of a training package for therapists
Source: BMJ Open. 2022 Jun 21;12(6):e056355. doi: 10.1136/bmjopen-2021-056355 (PMC9226877; doi:10.1136/bmjopen-2021-056355)
Supplement: Author's manuscript [file bmjopen-2021-056355.draft_revisions.pdf]

BMJ Open

BMJ Open is committed to open peer review. As part of this commitment we make the peer review history of every article we publish publicly available.

When an article is published we post the peer reviewers' comments and the authors' responses online. We also post the versions of the paper that were used during peer review. These are the versions that the peer review comments apply to.

The versions of the paper that follow are the versions that were submitted during the peer review process. They are not the versions of record or the final published versions. They should not be cited or distributed as the published version of this manuscript.

BMJ Open is an open access journal and the full, final, typeset and author-corrected version of record of the manuscript is available on our site with no access controls, subscription charges or pay-per-view fees (http://bmjopen.bmj.com).

If you have any questions on BMJ Open's open peer review process please email info.bmjopen@bmj.com

BMJ Open

Treating common mental disorder including psychotic experiences in the primary care Improving Access to Psychological Therapies programme (the TYPPEX study): protocol for a stepped wedge cluster randomised controlled trial with nested economic and process evaluation of a training package for therapists

Journal:	*BMJ Open*
Manuscript ID	bmjopen-2021-056355
Article Type:	Protocol
Date Submitted by the Author:	11-Aug-2021

Complete List of Authors:	Ashford, Polly-Anna; University of East Anglia, Department of Clinical Psychology and Psychological Therapies, Norwich Medical School<br>Knight, Clare; University of Cambridge Department of Psychiatry, Psychiatry;<br>Heslin, Margaret; Kings College London, King's Health Economics<br>Clark, Allan; Norwich Medical School<br>Kanaan, Mona; University of York, Health Sciences<br>Patel, Ushma; Cambridgeshire and Peterborough NHS Foundation Trust<br>Stuart, Freya; University of Cambridge Department of Psychiatry<br>Kabir, Thomas<br>Grey, Nick; Sussex Partnership NHS Foundation Trust; University of Sussex, Psychology<br>Murray, Hannah; University of Oxford,<br>Hodgekins , J ; Norwich Medical School, University of East Anglia, Norwich<br>Reeve, Nesta; Norfolk and Suffolk NHS Foundation Trust<br>Marshall, Nicola; Cambridgeshire and Peterborough NHS Foundation Trust<br>Painter, Michelle; Norfolk and Suffolk NHS Foundation Trust<br>Clarke, James; Cambridgeshire and Peterborough NHS Foundation Trust<br>Russo, Debra; University of Cambridge Department of Psychiatry<br>Stochl, Jan; University of Cambridge, Department of Psychiatry; NIHR Collaboration for Leadership in Applied Health Research & Care (CLAHRC) East of England,<br>Leathersich, Maria; University of East Anglia Norwich Medical School<br>Pond, Martin; University of East Anglia Norwich Medical School<br>Fowler, David; University of Sussex,<br>French, Paul; Manchester Metropolitan University<br>Swart, Ann Marie; University of East Anglia, Norwich Medical School<br>Dixon-Woods, Mary ; University of Cambridge Primary Care Unit, THIS Institute (The Healthcare Improvement Studies Institute)<br>Byford, Sarah; King's College London, Centre for the Economics of Mental and Physical Health<br>Jones, Peter; University of Cambridge, Department of Psychiatry<br>Perez, Jesus; Cambridgeshire and Peterborough NHS Foundation Trust

Keywords:	Anxiety disorders < PSYCHIATRY, Schizophrenia & psychotic disorders < PSYCHIATRY, PRIMARY CARE, Depression & mood disorders < PSYCHIATRY

SCHOLARONE™
Manuscripts

Treating common mental disorder including psychotic experiences in the primary care Improving Access to Psychological Therapies programme (the TYPPEX study): protocol for a stepped wedge cluster randomised controlled trial with nested economic and process evaluation of a training package for therapists

Polly-Anna Ashford,[1,*] Clare Knight,[2,*] Margaret Heslin,[3] Allan Clark,[1] Mona Kanaan,[4] Ushma Patel,[5] Freya Stuart,[2] Thomas Kabir,[6] Nick Grey,[7] Hannah Murray,[8] Joanne Hodgekins,[9] Nesta Reeve,[10] Nicola Marshall,[5] Michelle Painter,[10] James Clarke,[5] Debra A Russo,[2] Jan Stochl,[2] Maria Leathersich,[1] Martin Pond,[1] David Fowler,[11] Paul French,[12] Ann Marie Swart,[1] Mary Dixon-Woods,[13] Sarah Byford,[3] Peter B Jones[2,□] and Jesus Perez[5,□]

***Joint first authors**

□ Joint senior authors

1. Norwich Clinical Trials Unit, University of East Anglia, Norwich, UK.
2. Department of Psychiatry, University of Cambridge, Cambridge, UK.
3. King's College London, London, UK.
4. Department of Health Sciences, University of York, York, UK.
5. Cambridgeshire and Peterborough NHS Foundation Trust, Cambridge, UK.
6. The McPin Foundation, London, UK.
7. Sussex Partnership NHS Foundation Trust, Brighton, UK.
8. Oxford University, Oxford, UK.
9. Norwich Medical School, University of East Anglia, Norwich, UK.
10. Norfolk and Suffolk NHS Foundation Trust, Norwich, UK.
11. University of Sussex, Brighton, UK.
12. Manchester Metropolitan University, Manchester, UK
13. THIS Institute, University of Cambridge, Cambridge, UK.

Corresponding author: Jesus Perez

CAMEO Early Intervention Service, Chesterton Medical Centre, 35 Union Lane, Cambridge, CB41PX, Cambridge
Emails: jesus.perez@cpft.nhs.uk; jp440@cam.ac.uk
Phone: +44(0)1223458973

For peer review only

For peer review only - http://bmjopen.bmj.com/site/about/guidelines.xhtml

Abstract

Introduction

At least 1 in 4 people treated by the primary care Improving Access to Psychological Therapies (IAPT) programme in England experiences distressing psychotic experiences (PE) in addition to common mental disorder (CMD). These individuals are less likely to achieve recovery. IAPT services do not routinely screen for nor offer specific treatments for CMD including PE. The Tailoring evidence-based psychological therapY for People with common mental disorder including Psychotic EXperiences (TYPPEX) study will evaluate the clinical and cost-effectiveness of an enhanced training for cognitive behavioural therapists that aims to address this clinical gap.

Methods and analysis

This is a multisite, stepped-wedge cluster randomised controlled trial. The setting will be IAPT services within three mental health trusts. The participants will be (i) qualified IAPT cognitive behavioural therapists and (ii) service users who are triaged as appropriate for CBT in an IAPT service *and* have psychotic experiences according to the Community Assessment of Psychic Experiences - Positive 15-items Scale (CAPE-P15). IAPT therapists will be grouped into 8 study clusters subsequently randomised to the control-intervention sequence. We will obtain pseudonymous clinical outcome data from IAPT clinical records for eligible service users. We will invite service users to complete health economic measures at baseline, 3, 6, 9 and 12-month follow up. The primary outcome will be the proportion of patients with CMD-PE who have recovered by the end of treatment as measured by the official IAPT measure for recovery.

Ethics and dissemination

The study received the following approvals: South Central – Berkshire Research Ethics Committee on 28/04/2020 (REC reference 20/SC/0135) and HRA on 23/06/2020. An amendment was approved by the Ethics Committee on 01/10/2020 and HRA on 27/10/2020. Results will be made available to patients and the public, the funders, stakeholders in the IAPT services and other researchers.

Trial registration number

ISRCTN93895792

Keywords: Anxiety, depression, cognitive behavioural therapy, common mental disorders, IAPT, primary care, psychosis, psychotic experiences.

For peer review only

Strengths and limitations of this study

- The TYPPEX study will provide the first multisite, stepped-wedge cluster randomised controlled trial to investigate the clinical and cost-effectiveness of an intervention in the UK Improving Access to Psychological Therapies (IAPT) programme.
- The study intervention seeks to address the currently underserved needs of an important group of people accessing IAPT services. It further seeks to add to clinical understanding of psychotic experiences as markers for severity in common mental disorders.
- The stepped-wedge cluster design allows the intervention to be rolled out over time to all potential participants, while reducing operational and logistical challenges.
- The primary limitation is potential loss to follow-up, resulting in missing data for the secondary health economics outcome. This would challenge the internal validity of the conclusions drawn from the study.

Introduction

Psychotic experiences (PE), such as attenuated and/or fragmentary paranoid beliefs and hallucinations, are relatively common in the general population, especially amongst young people.[1] Though systematic reviews and evidence synthesis indicate that 30–40% of those with intense and frequent attenuated psychotic symptoms will transition to a psychotic disorder,[2, 3] studies including individuals with shorter and less intense mental symptoms have shown that far fewer (~10%) make such a conversion.[4, 5] Nonetheless, PE still predict propensity to seek treatment from mental health services[6] and are a marker for severity of other, non-psychotic common mental disorders (CMD), particularly depression and anxiety.[4, 7, 8]

In England, the Improving Access to Psychological Therapies (IAPT) programme is the main provision for people with common mental disorders. Offering UK National Institute for Health and Care Excellence (NICE)-approved psychological therapies, the programme is predominantly based on cognitive behavioural therapy (CBT). National standards specify that 50% of IAPT service users should achieve recovery, a key performance indicator defined by reduction in scores on indices of depression and anxiety. For the first time since its inception in 2008, IAPT met this target in 2016-17, with 51% of people nationally being considered recovered by the end of treatment.[9]

Evidence suggests that at least one in four people treated by IAPT may have a common mental disorder that includes psychotic experiences (CMD-PE). These individuals are at increased risk of not demonstrating recovery.[5, 10, 11] Currently, IAPT neither screens for, nor offers specific treatments for CMD-PE. Treatment protocols focus exclusively on mood

For peer review only

disturbance, leaving PE undetected and untreated. Given the complexity and co-morbidity of CMD-PE, standard treatment interventions are likely to be sub-optimal.

Though some effective psychological treatments for PE exist, they are dispersed throughout various service settings, and as such are inadequate to assess or treat the whole condition of CMD-PE. An innovative National Institute for Health Research (NIHR)-funded programme – Tailoring evidence-based psychological therapY for People with common mental disorder including Psychotic EXperiences (TYPPEX) – is targeting this clinical gap. It seeks to adapt and assemble available treatment options for CMD-PE into a practical therapeutic toolbox for cognitive behavioural therapists in IAPT known as CBT Tailoring for Severity (CBT-ts), rolled out via an enhanced training package and supervision programme.

This protocol paper describes the TYPPEX stepped wedge cluster Randomised Controlled Trial (TYPPEX swcRCT), which aims to determine the clinical and cost-effectiveness of CBT-ts versus IAPT standard care (SC) in IAPT service users with CMD and also PE, as determined by the Community Assessment of Psychic Experiences - Positive 15-items Scale (CAPE-P15)[12]. This pragmatic trial will be the first to identify and treat people with CMD-PE in IAPT services. It will provide valuable evidence for the development of interventions for this important and underserved population, while adding to the body of knowledge about design and conduct of randomised controlled trials in the IAPT setting.

Methods

This protocol is reported in accordance with Standard Protocol Items: Recommendations for Interventional Trials (SPIRIT) guidance[13] and refers to study protocol version 2.0 dated 4th of August 2020, which was the first version to be implemented. Amendments made to the protocol since commencement of the trial are shown in Table 1. Participant timelines and measures are summarised in Table 2.

-- Table 1 about here –

-- Table 2 about here –

Public and Patient Involvement

An experienced public and patient involvement (PPI) representative contributed significantly to the development of the PPI strategy set out in the grant application and continues to have study oversight as a member of the steering committee. The submission was endorsed by a co-applicant with relevant lived and academic experience and a PPI coordinator with lived experience was employed to facilitate PPI activity.

At the planning stage a Lived Experience Advisory Panel ('LEAP') was convened comprising 11 people with lived experience including diverse community members. The LEAP

For peer review only

influenced some research questions extending the scope of a systematic review within the research programme [14] and informing qualitative interview questions.

Since TYPPEX is a pragmatic trial, the study design is largely dictated according to the way that IAPT services operate. The primary outcome measure is a national metric of 'recovery' derived from routinely collected IAPT data, and is not open to alteration. LEAP members positively influenced secondary outcome measures including introducing the CAPE-P15 at final follow up.

LEAP involvement in the development of the intervention (CBT-ts) was highly influential, for example in review of the training resources and ideas about language and delivery. All patient-facing resources are produced in partnership with the LEAP, who have been invaluable in raising issues around acceptability and accessibility. The panel reviewed the feasibility study protocol and gave feedback on adaptations to remote working for the main trial. All ethical submissions undergo thorough PPI review with strong focus on acceptability of consent processes.

PPI representation from IAPT services was not initially possible since primary care services do not routinely screen for PE. However, after the trial's feasibility study, we have expanded involvement to include people recruited for such study, who have lived experience that matches our target population. We have also expanded involvement to include IAPT CBT therapists, also drawn from the feasibility study, to ensure acceptability and usability of CBT-ts and research methods within an IAPT setting, together contributing an additional seven perspectives.

People with lived experience will be involved in qualitative data interpretation to provide context and sense-checking of themes and anonymised service-user quotes as part of the process evaluation. LEAP members have co-authored papers and presented posters at public events and dissemination activity will continue to be informed and enhanced by people with lived experience.

PPI will remain reflective and responsive to new insights and the needs of the study, working to the NIHR INVOLVE's Standards for Public Involvement.[15]

Study Setting

The IAPT programme in England began in 2008 with the aim of improving access to evidence-based psychological treatment for CMD, particularly depression and anxiety. It sought to increase public access to National Institute for Health and Care Excellence (NICE)-approved psychological therapies, predominantly cognitive behavioural therapy (CBT), by offering flexible referral routes, including self-referral. The programme has continued to expand over time and currently assesses over 1,3 million people with CMD annually, delivering therapy to approximately 550,000.[9] NHS England has committed to further expansion of the programme, aiming to see 1.9 million patients annually by 2024.[16]·

For peer review only

The programme provides treatment for CMD for people aged 17 or over. Its pathways involve a stepped care model, providing steps 2 and 3 of the National Institute for Clinical Excellence (NICE) five-step approach to the treatment of CMD. At step 2 in the IAPT programme, large numbers of service users, with less severe conditions may be allocated to a single therapist. These service users may receive a variety of interventions, including brief face-to-face therapy, telephone support, computerised CBT or guided self-help. At step 3, highly trained CBT therapists manage smaller caseloads of people with more severe CMD. These therapists use more intensive treatment methods, delivering up to 20 individual face-to-face psychological therapy sessions. This clinical setting is immediately below psychiatric treatment by secondary mental health services (NICE steps 4 and 5).

Trial design and Participants

The TYPPEX study is a pragmatic, multi-centre stepped wedge cluster Randomised Controlled Trial (swcRCT) with nested health economic and process evaluations. It involves eight NICE step-3 IAPT teams (clusters) of CBT therapists across three mental health trusts in England: Cambridgeshire and Peterborough NHS Foundation Trust (CPFT), Norfolk and Suffolk NHS Foundation Trust (NSFT) and Sussex Partnership NHS Foundation Trust (SPFT). Each trust serves a mixed urban and rural population with a wide socio-economic range, including some high levels of deprivation, covering a total population of approximately 2.8 million.

Clusters will be randomised to different sequences rather than, strictly speaking, different arms. The sequences dictate the order in which each cluster switches from the control to intervention condition. TYPPEX swcRCT has five measurement periods and four steps, with two clusters switching to the intervention condition at each step. As the intervention is cluster randomised at the level of IAPT team, and the delivery of therapy by these teams may be subject to regional variation, each cluster will act as its own internal control. All clusters will start in the control condition and move to the intervention condition (see Figure 1).

The primary outcome of this study will be the proportion of eligible individuals with CMD-PE, as determined by the CAPE-P15 cut-off of 1.47[17], who have recovered by the end of treatment, measured via pseudonymous clinical outcome data obtained from IAPT clinical records and IAPT-defined metrics of recovery, as specified in Table 2.

-- Figure 1 about here –

Eligibility criteria

IAPT therapist inclusion criteria are: (1) qualified IAPT CBT therapist, and (2) willing and able to provide informed consent to receive CBT-ts training and supervision. IAPT therapist

For peer review only

exclusion criteria are: (1) does not deliver CBT therapy in current role, (2) works across more than one locality IAPT team, and (3) participated in an earlier TYPPEX feasibility study.

Service user participants are those individuals accepted onto the NICE step-3 IAPT caseload for participating therapists and meeting the study eligibility criteria, which are: (1) accepted onto the IAPT caseload for therapy and therefore meet service-specific inclusion criteria to access IAPT treatment (irrespective of whether service has been accessed previously or not), (2) meet current criteria for IAPT CBT treatment, and (3) assessed for PE, according to the CAPE-P15 questionnaire in their clinical record. Additional inclusion criteria for health economics sub-study only: (4) presence of PE according to the CAPE-P15 score, (5) in the judgement of the treating therapist has sufficient proficiency in English to complete research questionnaires, and (6) able and willing to provide written informed consent.

IAPT service user exclusion criteria are: (1) presence of mental disorder based on standard IAPT assessment meriting routine referral to NICE steps 4 or 5 treatment, i.e. to secondary mental health services.

Participant withdrawal

The right of any participant – therapist or service user – to discontinue participation without giving any reason will be respected. People will remain free to withdraw from the trial at any point without prejudicing their further treatment or, for therapists, employment. Reason for withdrawal, if given, will be recorded. Service users who have refused or withdrawn consent for data sharing will be excluded from pseudonymous routine data collection.

Randomisation

IAPT therapists from local teams within participating sites will be recruited and grouped as study clusters before randomisation (without stratification by cluster characteristics) to the control-intervention sequences via computer written code. Once clusters are allocated, IAPT service managers will be approached, and CBT-ts training courses arranged in line with each cluster randomisation point.

Blinding

The study will be conducted in a controlled open format with unblinded assessment; neither the research team nor the therapists that form the study clusters can be blind to the intervention. IAPT service users will be made aware of the study via an information leaflet given during screening but will not know whether their therapist has received CBT-ts training.

Sample size

CBT-ts training will be delivered to step-3 CBT therapists in eight clusters, randomised at four steps. In each cluster, approximately 7-10 therapists will receive training, with the intention for at least five therapists to deliver therapy throughout each cluster-period, to allow for inevitable therapist turnover and sickness. Based on our prevalence studies of

For peer review only

CMD-PE in IAPT [5, 10, 11] and considering average caseloads of 15-20 services users per therapist, each therapist will deliver therapy to a minimum of three people. All service users will be identified as having PE with the CAPE-P15 score threshold of 1.47[15] for both frequency of and distress associated to psychotic experiences at baseline, resulting in a minimum of 15 service users per cluster period. Eight clusters and four randomisation steps result in 20 control condition cluster-periods and 20 intervention condition cluster-periods. 300 IAPT service users will be recruited during the control condition and 300 during the intervention condition across all sites. In addition to the number of clusters and the steps presented above, we have assumed: an intra-cluster correlation coefficient of 0.05, a significance level of 0.05, and a difference of 0.39 and 0.58 between the control and intervention periods recovery. Based on these assumptions, the proposed sample size will allow us to detect such a difference with just over 80% power. Although this ignores that clustering also happens at the therapist level, it has been shown that ignoring an additional, lower level of clustering is a conservative estimate of the power. This is not sensitive to the choice of intra-cluster correlation coefficient [56].

In most cases, clusters will be formed from a single IAPT team. Some teams may have fewer IAPT CBT therapists available for training due to empty therapist posts or service demands. In these cases, two local IAPT teams will be merged to create a cluster. Pragmatically, the total number of service users scoring 1.47 and above on the CAPE-P15 and receiving therapy from a trained therapist during the whole trial may be higher, but only data from service users who complete therapy during a cluster period will be included in the primary outcome analysis. Based on results from our previous TYPPEX feasibility work, we expect that 6-month steps would be sufficient for this.

Also during the feasibility work we found that approximately 50% of the sample providing pseudonymised routine data would consent to complete the health economic measures. Thus, we aim that 150 service users will be consented to complete health economic measures during the control condition and 150 during the intervention phase across all sites.

Procedures

All IAPT service users who begin a course of CBT from the outset of the trial will be asked to complete a CAPE-P15 screening questionnaire and will receive a leaflet during their first treatment session. The leaflet explains that the trial is taking place in the service, and if eligible, their pseudonymous clinical data will be used for the research. Awareness of the trial is also raised in participating sites using posters. Both the poster and leaflet provide service users with information on the local policy for how they can opt out should they wish their data not to be used for research purposes. The CAPE-P15 screening questionnaire is currently accessible for clinical information as part of routine service use for participating sites.

For peer review only

IAPT service users with PE according to the CAPE-P15 will be asked by their therapist to provide consent to be contacted about participating in the health economic data collection, since this data cannot be collected from clinical records. If consent is obtained, the service user will be sent a participant information sheet and an electronic consent form by email (or paper versions by post) prior to the following treatment session, followed by baseline health economics measures.

Recruited service users will receive standard IAPT care from a participating therapist (control condition) or standard IAPT care from a participating therapist following CBT-ts training (intervention condition). They will be contacted by email or post for follow-up health economics data collection at 3, 6, 9, and 12 months after baseline.

Intervention

CBT-Tailoring for severity (CBT-ts)

CBT-ts is a team-level service improvement intervention. It aims to enhance existing CBT skills of NICE step-3 IAPT CBT therapists and is not a novel therapeutic intervention. Therefore, it is important to highlight that all IAPT service-users receiving CBT under the care of a CBT-ts trained therapist, including those who do not have CMD-PE, may be exposed to the effects of the training.

CBT-ts is based on the CBT framework currently used nationally in IAPT but includes adaptations to existing interventions to specifically address PE. The training takes a knowledge, skills and attitude focus enhancing existing knowledge of CBT. It includes three modules delivered as workshops supplemented by a blended learning package with access to an online network and provision of written resources. Therapists are provided with a single pack of editable templates, which they may or may not use in their clinical practice.

The structured training programme includes:

Module 1:

- Introduction to the concept of CMD-PE and the relationship between CMD and PE.
- Prevalence and impact in IAPT services.
- Identification of PE.
- Normalisation and validation of CMD-PE.

Module 2:

- Incorporating assessing PE into existing assessments within CBT.
- Suitability for IAPT.
- Understanding CMD-PE maintenance cycles.
- Approaches to the therapeutic conceptualisation and formulation of CMD-PE including co-morbidity.

For peer review only

Module 3:

- Adaptations to existing cognitive behavioural strategies and the application of these to promote the therapeutic change, using change methodologies, such as metaphors, exposure, cognitive restructuring and behavioural experiments.
- Relapse prevention.

Training is supported by six CBT-ts supervision sessions, which commence one month post training and are subsequently delivered monthly. Following completion of training, CBT therapists will identify people with CMD-PE according to the CAPE-P15, and treat them in line with current CBT interventions, incorporating their enhanced skills, as necessary. Treatment duration is non-prescriptive and expected to range between 8 to 14 sessions dependent upon therapists' clinical decisions. Treatment duration is not expected to exceed 20 weekly sessions, in line with current standard guidance in IAPT services, nationally.

Standard care

The control comparator is standard CBT care delivered within IAPT services prior to CBT-ts training. CBT is delivered as part of range of interventions offered at step 3 within the NICE stepped care model. CBT treatment protocols are delivered in accordance with NICE guidance for people with CMD. Treatment duration varies according to presenting problem and minimum dose necessary to achieve recovery, but NICE recommends individuals should receive up to 20 weekly sessions unless they recover beforehand (https://www.nice.org.uk/guidance/cg123/chapter/1-guidance).

Outcomes

A summary of study outcomes is shown in Table 2, and schedules of outcome measures for service users and therapists are provided in Tables 3 and 4.

-- Table 3 about here –

-- Table 4 about here –

Process evaluation

The aim of the process evaluation is to assess the views of all stakeholders involved in the experience and delivery of the TYPPEX swcRCT and CBT-ts, and to investigate the implementation of CBT-ts as designed, including any influences on uptake, delivery, and fidelity.

For peer review only

The aim of sampling for the process evaluation will be to achieve theoretical saturation [18], where further new data is unlikely to achieve further insights or to add to analytic depth. Up to 32 service users across the eight clusters with scores of 1.47 and above on the CAPE-P15 and who have completed at least two CBT treatment sessions with a CBT-ts trained therapist will be recruited including those who have completed treatment, those who are still undergoing therapy and those who dropped out of treatment. Where possible, purposive sampling will be used to ensure diversity of gender and ethnicity and exposure to number of sessions of therapy.

Between 16 and 24 IAPT therapists (with a minimum of two per cluster) who completed CBT-ts training and subsequently delivered at least three treatment sessions to a minimum of two service-users scoring 1.47 and above on the CAPE-P15 will be recruited. Additionally, we will conduct 18-25 interviews with IAPT clinical supervisors/managers from participating IAPT teams who line manage or supervise a CBT-ts trained therapist, senior non-clinical NHS managers with responsibility for delivery of IAPT services at Trust level or responsibility for commissioning IAPT services, and members of the trial team and collaborators who contributed to the design, development, or implementation of TYPPEX. In all cases, purposive sampling will be used to ensure representation of a wide range of perspectives and experiences.

Data management

Pseudonymised outcome data will be received by Norwich Clinical Trials Unit from sites and uploaded to a central study database stored on servers based at the University of East Anglia. Health economic questionnaire data will be collected using REDCap data collection software (https://www.project-redcap.org). Questionnaires will be completed online or by post by service users. Pseudonymised clinical data will be linked to consented participants' health economics data held by NCTU.

Data collection will include retrospectively recorded IAPT data from 23rd March 2020 until trial start. This data, covering the period of UK lockdown during the COVID-19 pandemic, will provide information on the nature of IAPT service provision during this time, and any changes in baseline morbidity. The aim of the data collection is to provide insight into standard of care and baseline severity during the pandemic, to assist decision-making by the trial oversight committees.

Data will be handled in accordance with a data management plan and comply with the principles of the International Conference on Harmonisation Good Clinical Practice, within the Standard Operating Procedures for Data Management in NCTU and where appropriate with UEA IT procedures. Interview transcriptions will be anonymised in adherence with the "Guidance on Anonymisation" issued by the UK Data Service (https://www.ukdataservice.ac.uk/manage-data/legal-ethical/anonymisation.aspx). Anonymised data will be stored in password-protected files on the firewall-protected University of Cambridge servers.

For peer review only - http://bmjopen.bmj.com/site/about/guidelines.xhtml

Statistical analyses

The primary analysis will compare IAPT-defined recovery of service users with CMD-PE as determined by the CAPE-P15 cut-off of 1.47 in the control phase with those in the intervention phase (post-training). Recovery proportions and means will be compared at individual therapist and team level after adjusting for the stepped-wedge cluster design. Adjustment will also be made for the baseline characteristics and other covariates. Time will be included as a set of indicator variables.

Analyses of clinical effectiveness will be based on the Intention to Treat (ITT) population with all available follow-up data from all new service users with CMD-PE on the caseload of participating therapists, controlling for baseline scores on depression and anxiety disorder specific measures. The primary ITT analysis is intended to provide inferences regarding the effectiveness of the intervention overall. It does not provide inferences regarding the causal effect of the intervention itself, but on the intervention as deployed in 'real life', therefore compliance information is not necessary to ensure that the ITT analysis is valid. As soon as a therapist has their first session of CBT-ts training, they begin to practise differently, so this date will mark the beginning of their intervention phase.

A logistic mixed effects regression model will be used for the primary outcome analysis. The cluster effect by both the therapist team and therapist will be considered in the multilevel analysis. Adjustment for potential prognostic variables will be agreed prior to analysis between the trial statisticians and the Chief Investigator.

Intra-cluster correlation coefficients and 95% confidence intervals will be reported for the primary and secondary outcomes. All analyses will be two-tailed and at the 5% level of significance.

Summary statistics of baseline characteristics at the cluster and the individual level will be reported where appropriate. Analyses will be conducted in Stata or R. Publication of results will include a participants/cluster flow diagram, and results will be reported according to the CONSORT statement for stepped wedge designs.

Economic analysis

The economic evaluation will be conducted covering the period from baseline to 12-month follow-up and will take the NHS/Personal Social Services perspective preferred by NICE[19]. The intervention will be costed taking a bottom-up (micro-costing) approach[20] using data collected on face-to-face contacts from IAPT records and accounting for the ratio of direct face-to-face to indirect non-face-to-face time. Data on indirect time, including preparation and supervision, will be collected directly from the therapists. Details of resources required to deliver the CBT-ts training and supervision will be provided by the TYPPEX research team. Use of all other health and social care services will be collected via a modified version of the Adult Service Use Schedule for Early Intervention (EI-ADSUS; Table 1) collected using REDCap data collection software at baseline (covering the last 3 months) and 3-, 6-, 9-, and

For peer review only

12-months (covering the period since the last interview). Resource use data will be combined with nationally applicable unit costs.[21, 22] Productivity losses (applicable to sensitivity analyses only) will be calculated using the human capital approach by multiplying days off work for mental health reasons by the national average wage rate.[23]

Our primary economic analysis will be a cost-utility analysis using Quality Adjusted Life Years (QALYs) derived from the EuroQol measure of health-related quality of life (EQ-5D) based on complete case data. The EQ-5D-5L has been proposed as being more sensitive to change in mental health populations compared to the EQ-5D-3L.[24] However, there is little evidence to support this hypothesis. Therefore, we will compare the psychometric properties of the 3L and 5L versions. The version of the EQ-5D used in this analysis will be dependent on analyses comparing the two. QALYs will be calculated by applying appropriate utility weights to EQ-5D health states[25] and using the total area under the curve approach with linear interpolation between assessment points.[26] A secondary analysis will additionally explore cost-effectiveness using the primary outcome measure: IAPT-defined recovery.

Costs and outcomes will be compared at the final follow-up point and presented as mean values by trial phase with standard deviations. Mean differences in costs and 95% confidence intervals will be obtained by nonparametric bootstrap regressions to account for the non-normal distribution commonly found in economic data[27]. Incremental cost-effectiveness ratios (ICERs) will be calculated if higher costs and better outcomes are found in either the intervention or control group. Uncertainty will be explored using cost-effectiveness planes and cost-effectiveness acceptability curves (CEACs) based on the net-benefit approach.[28] These curves are an alternative to confidence intervals around incremental cost-effectiveness ratios and show the probability that one intervention is cost effective compared to the other for a range of values that a decision maker would be willing to pay for an additional unit of an outcome. To provide more relevant treatment-effect estimates, all economic analyses will include adjustment for stepped-wedge cluster design, relevant baseline characteristics and other covariates, in line with the clinical analyses.[29]

Sensitivity analyses will be conducted to explore the impact of missing data and to explore cost-effectiveness from a broader perspective, including productivity losses because of time off work due to illness.

Process evaluation analysis

Analysis of process evaluation data will be based on the constant comparative method.[30, 31] Practically, this will be achieved through multiple levels of analysis, systematically comparing data on different questions and from different sources (quantitative and qualitative) to achieve thematically ordered synthesis. Data analysis will take place after each stage of data collection so the analysis from each stage can inform data collection in the next. Some data analysis will also take place in parallel. The data will initially be subject to open coding using a combination of pre-selected questions and sensitising constructs identified from the literature. Codes will be increasingly grouped into higher-order explanatory categories through comparison and refinement in rounds of discussion and

For peer review only

sense-making. A set of overarching thematic categories will be agreed and used as a framework for further, more deductive, coding across the whole data set.

Trial Status

The trial was opened to IAPT service user recruitment in March 2021, with the first randomisation point scheduled for September 2021. Recruitment will continue until September 2023. The final 12 month follow-up is expected to be completed by October 2024.

Ethics and dissemination

This study was approved by the South Central – Berkshire Research Ethics Committee (REC reference 20/SC/0135 on 28/04/2020 and HRA on 23/06/2020). Subsequent amendments are shown in Table 1.

The study is jointly sponsored by the University of Cambridge and Cambridgeshire and Peterborough NHS Foundation Trust. It was adopted onto the NIHR trial portfolio on 16/04/2020.

CBT-ts, including therapist training and supervision, is low risk for all participants. IAPT service users scoring above the CAPE-P15 thresholds for PE on whom data is being pseudonymously collected are under the care of the IAPT service for the duration of their participation and are subject to normal IAPT safety procedures. No specific risks, untoward incidents or adverse events related to CBT-ts are anticipated as the intervention aims to up-skill therapists rather than offering a new therapeutic intervention. This approach was supported by the results of a feasibility study as part of the wider TYPPEX research programme, during which no related serious adverse events were reported.

Safety outcome variables will be collected retrospectively each month from the routinely collected pseudonymous data. Aggregated safety information will be reported to the TYPPEX Data Monitoring and Ethics Committee (DMEC) throughout the randomisation condition. In addition, Principal Investigators, IAPT managers and therapists will be requested to report complaints relating to TYPPEX by eligible participants to the trial team.

Except for the health economic questionnaires (EI-ADSUS, EQ-5D-5L and EQ-5D-3L), all data for the trial are collected as part of routine care. Therefore, there is no direct recruitment of service users for the primary outcome; anonymised data collection with pseudonymous linkage to consented health economic questionnaires for a sub-group of service users is designed to remove the burden on IAPT therapists with respect to discussing the trial, and to ensure a representative sample.

Results from this trial will be presented at national and international conferences, including the Schizophrenia International Research Society (SIRS) Conference and IEPA *Early Intervention in Mental Health* Conference. Dissemination will also occur through the

For peer review only

submission of a primary article on the outcomes and several subsequent articles considering the effects of the intervention stratified by service user and therapist variables. Primary outcomes will be disseminated more widely as part of a theory-driven implementation plan, developing a community of practise (CoP) including patients, the public, economists and systems engineers experienced in care pathway design and evaluation, in addition to stakeholders in the IAPT services.

The training materials will be shared electronically and will be widely available to the NHS.

Contributors

PBJ and JP are co-CIs for and designed this trial. P-AA is the trial manager. CK was manager for the wider research programme. UP led the design and development of the intervention, supported by DAR. JH, NR, NM, JC, MP, NG, HM, DF and PF advised on the development of the intervention. TK leads on the PPI strategy. FS supports all elements of the PPI strategy and, with DAR, also delivers research assistant functions for the trial. AC and MK contributed to the trial design and oversee the statistical analysis. JS advises on statistical matters. MH and SB lead the health economic component of the trial. MD-W oversees qualitative analyses and process evaluation. ML and MP are responsible for trial data management. AMS oversees all operations at the Clinical Trials Unit. P-AA, CK, MH, AC, MK and JP wrote the first draft of this manuscript. All authors reviewed, contributed to, and commented on the final draft.

Funding

This paper presents independent research funded by the National Institute for Health Research (NIHR) under its Programme Grants for Applied Research Programme (Reference Number RP-PG-0616-20003). The views expressed are those of the author(s) and not necessarily those of the NHS, the NIHR or the Department of Health.

The funding body had no role in study design; collection, analysis, or interpretation of data; the writing of the report; or decision to submit the paper. The first author had full access to all data and had final responsibility for the decision to submit for publication.

Competing interests

None declared.

For peer review only

References

1. Linscott RJ, van Os J. An updated and conservative systematic review and meta-analysis of epidemiological evidence on psychotic experiences in children and adults: on the pathway from proneness to persistence to dimensional expression across mental disorders. *Psychol Med*2013;43(6):1133-49 doi:10.1017/S0033291712001626.

2. Fusar-Poli P, Bonoldi I, Yung AR, Borgwardt S, et al. Predicting psychosis: meta-analysis of transition outcomes in individuals at high clinical risk. *Arch Gen Psychiatry*2012;69(3):220-9 doi:10.1001/archgenpsychiatry.2011.1472.

3. Addington J, Epstein I, Liu L, et al. A randomized controlled trial of cognitive behavioral therapy for individuals at clinical high risk of psychosis. *Schizophr Res*2011;125(1):54-61 doi:10.1016/j.schres.2010.10.015.

4. Hui, C, Morcillo, C, Russo, DA, et al. Psychiatric morbidity, functioning and quality of life in young people at clinical high risk for psychosis. *Schizophr Res* 2013;148(1–3):175–180.

5. Perez J, Russo DA, Stochl J, et al. Common mental disorder including psychotic experiences: trailblazing a new recovery pathway within the Improving Access to Psychological Therapies programme in England. *Early Interv Psychia*2017;12(3):497–504 doi:https://doi.org/10.1111/eip.12434.

6. Devylder JE, Oh HY, Corcoran CM, et al. Treatment seeking an unmet need for care among persons reporting psychosis-like experiences. *Psychiatr Serv*2014;65(6):774–780 doi:https://doi.org/10.1176/appi.ps.201300254.

7. Wigman JT, Nierop M.V, Vollebergh WA, et al. Evidence that psychotic symptoms are prevalent in disorders of anxiety and depression, impacting on illness onset, risk, and severity–implications for diagnosis and ultra-high risk research. *Schizophr Bull*2012;38(2):247–257 doi:https://doi.org/10.1093/schbul/sbr196.

8. Stochl J, Khandaker GM, Lewis G, et al. Mood, anxiety and psychotic phenomena measure a common psychopathological factor. *Psychol Med*2015;45(7):1–11. doi:10.1017/S003329171400261X.

9. NHS Digital. Psychological Therapies, Annual report on the use of IAPT services - England, 2017-18 (online). https://digital.nhs.uk/data-and-information/publications/statistical/psychological-therapies-annual-reports-on-the-use-of-iapt-services/annual-report-2017---18 (accessed September 2020).

10. Perez J, Jin H, Russo D, et al. Improving the detection of individual at risk of developing psychosis in primary mental health care. *Schizophr Bull* 2018:44;S57.

11. Knight C, Russo D, Stochl J, et al. Prevalence of and recovery from common mental disorder including psychotic experiences in the UK Primary Care Improving Access to Psychological Therapies (IAPT) Programme. *J Affect Disord*2020;(1):272:84-90 doi:10.1016/j.jad.2020.04.015.

12. Capra C, Kavanagh DJ, Hides L, et al. Current CAPE-15: a measure of recent psychotic-like experiences and associated distress. *Early Interv Psychia*2017;11(5):411–417 doi: https://doi.org/10.1111/eip.12245.

13. Chan A-W, Tetzlaff JM, Altman DG, et al. SPIRIT 2013 Statement: Defining standard protocol items for clinical trials. *Ann Intern Med* 2013;158(3):200-207.

14. Soneson E, Russo D, Stochl J, et al. Psychological interventions for people with psychotic experiences: A systematic review and meta-analysis of controlled and uncontrolled effectiveness and economic studies. *Aust N Z J Psychiatry* 2020;54(7):673-695. doi: 10.1177/0004867420913118.

15. NIHR Involve. National Standards for Public Involvement in Research V1 March 2018 (online). https://www.invo.org.uk/posttypepublication/national-standards-for-public-involvement/ (accessed November 2020).

16. Clark DM. IAPT at 10: achievements and challenges, 2019 (online). https://www.england.nhs.uk/blog/iapt-at-10-achievements-and-challenges/ (accessed September 2019).

17. Bukenaite A, Stochl J, Mossaheb N, et al. Usefulness of the CAPE-P15 for detecting people at ultra-high risk for psychosis: Psychometric properties and cut-off values. *Schizophr Res* 2017;189:69-74. doi: 10.1016/j.schres.2017.02.017.

18. Guest G, Namey E, Chen M. A simple method to assess and report thematic saturation in qualitative research. PLoS One. 2020;15(5):e0232076

19. National Institute for Health and Care Excellence. Guide to the methods of technology appraisal 2013. London: NICE 2013.

20. Drummond MF, Sculpher MJ, Torrance GW, et al. Methods for the economic evaluation of health care programmes. New York: Oxford University Press 2005.

21. Curtis L, Burns A. Unit Costs of Health and Social Care 2018. University of Kent, Canterbury: Personal Social Services Research Unit 2018.

22. NHS improvement. 2017/18 Reference Costs (online). https://webarchive.nationalarchives.gov.uk/20200501111106/https://improvement.nhs.uk/documents/6468/201718_reference_costs_data_and_guidance.zip/ (accessed March 2020).

23. Koopmanschap MA, Rutten FF. A practical guide for calculating indirect costs of disease. *Pharmacoeconomics*1996 Nov;10(5):460-6. doi:10.2165/00019053-199610050-00003.

24. Camacho EM, Shields G, Lovell K, et al. A (five-) level playing field for mental health conditions?: exploratory analysis of EQ-5D-5L-derived utility values. *Qual Life Res* 2018;27(3):717-24.

25. Dolan P, Gudex C, Kind P, et al. A social tariff for EuroQol: results from a UK general population survey. York: University of York, Centre for Health Economics 1995.

26. Manca A, Hawkins N, Schulpher MJ. Estimating mean QALYs in trial-based cost-effectiveness analysis: the importance of controlling for baseline utility. *Health Econ* 2005;14(5): 487-496.

27. Thompson SG, Barber JA. How should cost data in pragmatic randomised trials be analysed? *BMJ*. 2000;320:1197-200.

28. Briggs AH. A Bayesian approach to stochastic cost-effectiveness analysis. *Health Econ* 1999;8:257-261.

29. Assmann SF, Pocock SJ, Enos LE, et al. Subgroup analysis and other (mis) uses of baseline data in clinical trials. *Lancet*. 2000 Mar 25;355(9209):1064-9.

30. Charmaz K. Constructing grounded theory. London: Sage Publications 2014.

For peer review only

31. Charmaz K. Grounded theory: Objectivist and constructivist methods. In: Denzin NK, Lincoln YS, eds. Strategies for qualitative inquiry (2nd ed.). Thousand Oaks, CA: Sage; 2003:249-291.

32. Kroenke K, Spitzer RL, Williams JB. The PHQ-9: validity of a brief depression severity measure. *J Gen Intern Med* 2001;16(9):606–613. doi:10.1046/j.1525-1497.2001.016009606.x.

33. Spitzer RL, Kroenke K, Williams JB, et al. A brief measure for assessing generalized anxiety disorder: the GAD-7. *Arch Intern Med* 2006;166(10):1092–1097 doi:https://doi.org/10.1001/archinte.166.10.1092.

34. Perez J, Jin H, Russo DA, et al. Clinical and cost-effectiveness of tailored, intensive liaison between primary and secondary care to detect individuals at risk of a first psychotic illness: a theory-based, cluster-randomised controlled trial. *Lancet Psychiatry*. 2015;2(11):984-93.

35. The National Collaborating Centre for Mental Health, 2018. The improving access to psychological therapies manual (online). https://www.rcpsych.ac.uk/docs/default-source/improving-care/nccmh/the-iapt-manual–final–republished-7-3-18.pdf?sfvrsn= a192d1af_0 (accessed September 2020).

36. Knight C, Russo D, Stochl J, et al. More sensitive identification of psychotic experiences in common mental disorder by primary mental healthcare services - effect on prevalence and recovery: casting the net wider. *BJPsych Open* 2020;6(6):e136.

37. Group TE. EuroQol-a new facility for the measurement of health-related quality of life. *Health Policy* 1990;16(3):199-208.

38. Herdman M, Gudex C, Lloyd A, et al. Development and preliminary testing of the new five-level version of EQ-5D (EQ-5D-5L). *Qual Life Res* 2011;20(10):1727-36.

39. Stochl J, Croudace T, Perez J, et al. Usefulness of EQ-5D for evaluation of health-related quality of life in young adults with first-episode psychosis. *Qual Life Res* 2013;22(5):1055-63.

40. Lovett R, Cooper S, NICE Science policy and research programme. NICE to support new valuation study for England for EQ-5D-5L questionnaire (online). https://www.nice.org.uk/news/blog/nice-to-support-new-valuation-study-for-england-for-eq-5d-5l-questionnaire (accessed May 2021).

For peer review only

Figure 1: Stepped wedge trial diagram

Cluster	RANDOMISATION	Control	Step 1	Step 2	Step 3	Step 4
1						I
2						I
3					I	I
4					I	I
5				I	I	I
6				I	I	I
7			I	I	I	I
8			I	I	I	I
		Months 1-6	Months 7-12	Months 13-18	Months 19-24	Months 25-30

Key:

Control phase	(grey)
Intervention phase (I)	(blue)

For peer review only

Table 1: Summary of ethical amendments

Protocol	Primary reasons for amendment
2.0, 04/08/2020	• Addition of secondary clinical outcomes (Reliable Recovery and Reliable Improvement).<br>• Addition of retrospective pseudonymous clinical data collection from the beginning of UK lockdown due to COVID-19, until randomisation.<br>• Intervention adaptation: training to be delivered either online or face-to-face.<br>• Changes to the service user consent process: moving from full consent on tablet devices to consent to contact followed by full consent online at home.
3.0, 12/05/2021	• Video conferencing e.g. Zoom included as an option for Process Evaluation interviews.<br>• Removal of restriction of only teams from the same NHS Trust merging to form a cluster.

For peer review only

Table 2: Summary of study outcomes

Category	Measure	Primary/Secondary	Details
Service User Recovery	IAPT-defined 'Recovery'	Primary	The primary outcome is the proportion of IAPT service-users with CAPE-P15 score of 1.47 or above who have recovered by the end of treatment. Recovery is a national IAPT programme performance metric, with an individual deemed recovered if they scored above the clinical cut-off on the PHQ-9[32] and/or GAD-7[33] before treatment, i.e. 10 and 8 respectively, and below the clinical cut-off at the end of treatment on both measures.[34] If recorded, an anxiety disorder specific measure (ADSM) is used in place of the GAD-7 if that is the focus of treatment.[35]
	IAPT-defined 'Recovery'	Secondary	As for the primary outcome, but for IAPT service-users reaching the lower threshold score for CMD-PE (CAPE-P15 score of 1.30 and above).[36]
	IAPT-defined 'Reliable Improvement'	Secondary	Service users are considered reliably improved if they show any improvement in scores on the appropriate outcome measures between pre and post treatment, that exceeds the measurement error of the scales.[35]
	IAPT-defined 'Reliable Recovery'	Secondary	Service users are considered reliably recovered if they meet both criteria for Reliable Improvement and for Recovery.[35]
Health economic measures	Modified Adult Service Use Schedule for Early Intervention (EI-ADSUS)	Secondary	Individual-level resource use will be measured using a modified version of Adult Service Use Schedule for Early Intervention (EI-ADSUS). This was developed in previous research with similar populations[34] and adapted for electronic participant self-completion. It will include all-cause hospital and community-based health and social care resource use as well as information on time off work due to mental health.
	EuroQol measure of health-related quality of life 3 level (EQ-5D-3L)	Secondary	Health related quality of life will be measured using the EQ-5D-3L, a generic, preference-based measure based on five dimensions (mobility, self-care, usual activities, pain/discomfort, and anxiety/depression). Each dimension is rated on three levels (no problems, some problems, and severe problems).[37]
	EuroQol measure of health-related	Secondary	The EQ-5D-5L is also a health-related quality of life measure based on five dimensions (mobility, self-care, usual activities, pain/discomfort and

For peer review only

	quality of life 5 level (EQ-5D-5L)		anxiety/depression), but each dimension is rated on five levels (no problems, slight problems, moderate problems, severe problems, and extreme problems.[38]<br><br>It has been demonstrated that the EQ-5D-3L is a useful, valid instrument in young people with emerging psychotic symptoms.[39] However, the new five-level version, may prove more sensitive to change and is now recommended for economic evaluation by NICE[40] but has not been validated in this population. Therefore, we will test the sensitivity of the EQ-5D-5L in comparison to the EQ-5D-3L.
Therapist Adherence	Supervision checklist	Secondary	Therapist adherence measured using a supervision checklist and adherence score completed during the 6 monthly supervision sessions held after CBT training. Trial supervisors will review and collate therapy session notes and rate adherence to the CBT-ts approach.

For peer review only

Table 3: Schedule of IAPT user enrolment, interventions, and assessments.

	**STUDY PERIOD (CONTROL AND RANDOMISED CONDITIONS)**						
	Screening	Baseline	Treatment	Follow Up			
**TIMEPOINT**	**-t1***	**-t2**	**Up to 20 sessions**	**3**	**6**	**9**	**12**
**ENROLMENT**							
Eligibility screen	x						
Consent to contact (for health economics measures only)							
Informed consent (for health economics measures only)		x					
IAPT CBT standard care							
**ASSESSMENTS**							
PHQ-9	x	x	X				x
GAD-7/ADSM	x	x	X				x
EQ-5D-3L		x		x	x	x	x
EQ-5D-5L		x		x	x	x	x
EI-ADSUS		x		x	x	x	x
CAPE P-15							x

*The duration between the screening visit and baseline is anticipated to be 1-3 weeks, depending on typical frequency of therapy sessions in IAPT service. During this time the CAPE-P15 is scored and eligibility is confirmed. CAPE+ IAPT users are approached to participate in the health economics data collection during the baseline visit. Clinical data is obtained for all CAPE+ IAPT users unless they opt out.

**PHQ-9 and GAD-7 are routine IAPT clinical measures and are collected during every clinical contact.

For peer review only

Table 4: Schedule of IAPT therapist enrolment, interventions, and assessments.

	Trial set-up	Randomisation	Control condition	Intervention condition
**IDENTIFICATION**				
IAPT teams and potential therapists identified and grouped to form clusters	x			
**ENROLMENT AND RANDOMISATION**				
Informed consent	x			
Clusters randomised		x		
**INTERVENTION**				
CBT-ts training				x
CBT-ts supervision				x
**ASSESSMENTS**				
Adherence and engagement record				x

For peer review only

STANDARD PROTOCOL ITEMS: RECOMMENDATIONS FOR INTERVENTIONAL TRIALS

SPIRIT 2013 Checklist: Recommended items to address in a clinical trial protocol and related documents*

Section/item	Item No	Description	Addressed on page number
**Administrative information**			
Title	1	Descriptive title identifying the study design, population, interventions, and, if applicable, trial acronym	________1_____
Trial registration	2a	Trial identifier and registry name. If not yet registered, name of intended registry	________2_____
	2b	All items from the World Health Organization Trial Registration Data Set	________2_____
Protocol version	3	Date and version identifier	________4_____
Funding	4	Sources and types of financial, material, and other support	________15_____
Roles and responsibilities	5a	Names, affiliations, and roles of protocol contributors	______15______
	5b	Name and contact information for the trial sponsor	________14_____
	5c	Role of study sponsor and funders, if any, in study design; collection, management, analysis, and interpretation of data; writing of the report; and the decision to submit the report for publication, including whether they will have ultimate authority over any of these activities	_____n/a_______
	5d	Composition, roles, and responsibilities of the coordinating centre, steering committee, endpoint adjudication committee, data management team, and other individuals or groups overseeing the trial, if applicable (see Item 21a for data monitoring committee)	_____n/a_______

For peer review only - http://bmjopen.bmj.com/site/about/guidelines.xhtml

Introduction

Background and rationale	6a	Description of research question and justification for undertaking the trial, including summary of relevant studies (published and unpublished) examining benefits and harms for each intervention	____3_________
	6b	Explanation for choice of comparators	____6_________
Objectives	7	Specific objectives or hypotheses	____4_________
Trial design	8	Description of trial design including type of trial (eg, parallel group, crossover, factorial, single group), allocation ratio, and framework (eg, superiority, equivalence, noninferiority, exploratory)	____6_________

Methods: Participants, interventions, and outcomes

Study setting	9	Description of study settings (eg, community clinic, academic hospital) and list of countries where data will be collected. Reference to where list of study sites can be obtained	_____5________
Eligibility criteria	10	Inclusion and exclusion criteria for participants. If applicable, eligibility criteria for study centres and individuals who will perform the interventions (eg, surgeons, psychotherapists)	_____6________
Interventions	11a	Interventions for each group with sufficient detail to allow replication, including how and when they will be administered	_____9________
	11b	Criteria for discontinuing or modifying allocated interventions for a given trial participant (eg, drug dose change in response to harms, participant request, or improving/worsening disease)	_____n/a_______
	11c	Strategies to improve adherence to intervention protocols, and any procedures for monitoring adherence (eg, drug tablet return, laboratory tests)	_______10______
	11d	Relevant concomitant care and interventions that are permitted or prohibited during the trial	_______n/a______
Outcomes	12	Primary, secondary, and other outcomes, including the specific measurement variable (eg, systolic blood pressure), analysis metric (eg, change from baseline, final value, time to event), method of aggregation (eg, median, proportion), and time point for each outcome. Explanation of the clinical relevance of chosen efficacy and harm outcomes is strongly recommended	_______10_______
Participant timeline	13	Time schedule of enrolment, interventions (including any run-ins and washouts), assessments, and visits for participants. A schematic diagram is highly recommended (see Figure)	______10_______

For peer review only - http://bmjopen.bmj.com/site/about/guidelines.xhtml

Sample size	14	Estimated number of participants needed to achieve study objectives and how it was determined, including clinical and statistical assumptions supporting any sample size calculations	_____7________
Recruitment	15	Strategies for achieving adequate participant enrolment to reach target sample size	_____8________

Methods: Assignment of interventions (for controlled trials)

Allocation:

Sequence generation	16a	Method of generating the allocation sequence (eg, computer-generated random numbers), and list of any factors for stratification. To reduce predictability of a random sequence, details of any planned restriction (eg, blocking) should be provided in a separate document that is unavailable to those who enrol participants or assign interventions	___7__________
Allocation concealment mechanism	16b	Mechanism of implementing the allocation sequence (eg, central telephone; sequentially numbered, opaque, sealed envelopes), describing any steps to conceal the sequence until interventions are assigned	___7_________
Implementation	16c	Who will generate the allocation sequence, who will enrol participants, and who will assign participants to interventions	____8_________
Blinding (masking)	17a	Who will be blinded after assignment to interventions (eg, trial participants, care providers, outcome assessors, data analysts), and how	___n/a________
	17b	If blinded, circumstances under which unblinding is permissible, and procedure for revealing a participant's allocated intervention during the trial	____n/a_______

Methods: Data collection, management, and analysis

Data collection methods	18a	Plans for assessment and collection of outcome, baseline, and other trial data, including any related processes to promote data quality (eg, duplicate measurements, training of assessors) and a description of study instruments (eg, questionnaires, laboratory tests) along with their reliability and validity, if known. Reference to where data collection forms can be found, if not in the protocol	___4,11,12_____
	18b	Plans to promote participant retention and complete follow-up, including list of any outcome data to be collected for participants who discontinue or deviate from intervention protocols	____7________

Data management	19	Plans for data entry, coding, security, and storage, including any related processes to promote data quality (eg, double data entry; range checks for data values). Reference to where details of data management procedures can be found, if not in the protocol	____11________
Statistical methods	20a	Statistical methods for analysing primary and secondary outcomes. Reference to where other details of the statistical analysis plan can be found, if not in the protocol	_____12_______
	20b	Methods for any additional analyses (eg, subgroup and adjusted analyses)	_____12_______
	20c	Definition of analysis population relating to protocol non-adherence (eg, as randomised analysis), and any statistical methods to handle missing data (eg, multiple imputation)	_____12_______
**Methods: Monitoring**			
Data monitoring	21a	Composition of data monitoring committee (DMC); summary of its role and reporting structure; statement of whether it is independent from the sponsor and competing interests; and reference to where further details about its charter can be found, if not in the protocol. Alternatively, an explanation of why a DMC is not needed	_____14_______
	21b	Description of any interim analyses and stopping guidelines, including who will have access to these interim results and make the final decision to terminate the trial	____-________
Harms	22	Plans for collecting, assessing, reporting, and managing solicited and spontaneously reported adverse events and other unintended effects of trial interventions or trial conduct	_____14_______
Auditing	23	Frequency and procedures for auditing trial conduct, if any, and whether the process will be independent from investigators and the sponsor	______-______
**Ethics and dissemination**			
Research ethics approval	24	Plans for seeking research ethics committee/institutional review board (REC/IRB) approval	____2________
Protocol amendments	25	Plans for communicating important protocol modifications (eg, changes to eligibility criteria, outcomes, analyses) to relevant parties (eg, investigators, REC/IRBs, trial participants, trial registries, journals, regulators)	_____4_______

For peer review only - http://bmjopen.bmj.com/site/about/guidelines.xhtml

Consent or assent	26a	Who will obtain informed consent or assent from potential trial participants or authorised surrogates, and how (see Item 32)	_____8_______
	26b	Additional consent provisions for collection and use of participant data and biological specimens in ancillary studies, if applicable	_____n/a______
Confidentiality	27	How personal information about potential and enrolled participants will be collected, shared, and maintained in order to protect confidentiality before, during, and after the trial	______11______
Declaration of interests	28	Financial and other competing interests for principal investigators for the overall trial and each study site	______15______
Access to data	29	Statement of who will have access to the final trial dataset, and disclosure of contractual agreements that limit such access for investigators	______11_______
Ancillary and post-trial care	30	Provisions, if any, for ancillary and post-trial care, and for compensation to those who suffer harm from trial participation	______-_______
Dissemination policy	31a	Plans for investigators and sponsor to communicate trial results to participants, healthcare professionals, the public, and other relevant groups (eg, via publication, reporting in results databases, or other data sharing arrangements), including any publication restrictions	_____14_______
	31b	Authorship eligibility guidelines and any intended use of professional writers	______-______
	31c	Plans, if any, for granting public access to the full protocol, participant-level dataset, and statistical code	_____-_______
**Appendices**			
Informed consent materials	32	Model consent form and other related documentation given to participants and authorised surrogates	__on request_
Biological specimens	33	Plans for collection, laboratory evaluation, and storage of biological specimens for genetic or molecular analysis in the current trial and for future use in ancillary studies, if applicable	_____n/a______

*It is strongly recommended that this checklist be read in conjunction with the SPIRIT 2013 Explanation & Elaboration for important clarification on the items. Amendments to the protocol should be tracked and dated. The SPIRIT checklist is copyrighted by the SPIRIT Group under the Creative Commons "Attribution-NonCommercial-NoDerivs 3.0 Unported" license.

BMJ Open

Treating common mental disorder including psychotic experiences in the primary care Improving Access to Psychological Therapies programme (the TYPPEX study): protocol for a stepped wedge cluster randomised controlled trial with nested economic and process evaluation of a training package for therapists

Journal:	*BMJ Open*
Manuscript ID	bmjopen-2021-056355.R1
Article Type:	Protocol
Date Submitted by the Author:	27-Apr-2022

Complete List of Authors:	Ashford, Polly-Anna; University of East Anglia, Department of Clinical Psychology and Psychological Therapies, Norwich Medical School<br>Knight, Clare; University of Cambridge Department of Psychiatry, Psychiatry<br>Heslin, Margaret; King's College London Institute of Psychiatry Psychology and Neuroscience, King's Health Economics<br>Clark, Allan; University of East Anglia, Norwich Medical School<br>Kanaan, Mona; University of York, Health Sciences<br>Patel, Ushma; Cambridgeshire and Peterborough NHS Foundation Trust<br>Stuart, Freya; University of Cambridge Department of Psychiatry<br>Kabir, Thomas; The McPin Foundation<br>Grey, Nick; Sussex Partnership NHS Foundation Trust; University of Sussex, Psychology<br>Murray, Hannah; University of Oxford,<br>Hodgekins , J ; University of East Anglia, Norwich Medical School<br>Reeve, Nesta; Norfolk and Suffolk NHS Foundation Trust<br>Marshall, Nicola; Cambridgeshire and Peterborough NHS Foundation Trust<br>Painter, Michelle; Norfolk and Suffolk NHS Foundation Trust<br>Clarke, James; Cambridgeshire and Peterborough NHS Foundation Trust<br>Russo, Debra; University of Cambridge Department of Psychiatry<br>Stochl, Jan; University of Cambridge, Department of Psychiatry<br>Leathersich, Maria; University of East Anglia Norwich Medical School<br>Pond, Martin; University of East Anglia Norwich Medical School<br>Fowler, David; University of Sussex,<br>French, Paul; Manchester Metropolitan University<br>Swart, Ann Marie; University of East Anglia, Norwich Medical School<br>Dixon-Woods, Mary ; University of Cambridge Primary Care Unit, THIS Institute (The Healthcare Improvement Studies Institute)<br>Byford, Sarah; King's College London, Centre for the Economics of Mental and Physical Health<br>Jones, Peter; University of Cambridge, Department of Psychiatry<br>Perez, Jesus; Cambridgeshire and Peterborough NHS Foundation Trust; IBSAL, Medicine, Universidad de Salamanca

**Primary Subject Heading**:	Mental health
Secondary Subject Heading:	Health services research, Patient-centred medicine, General practice / Family practice
Keywords:	Anxiety disorders < PSYCHIATRY, Schizophrenia & psychotic disorders < PSYCHIATRY, PRIMARY CARE, Depression & mood disorders < PSYCHIATRY

SCHOLARONE™
Manuscripts

Treating common mental disorder including psychotic experiences in the primary care Improving Access to Psychological Therapies programme (the TYPPEX study): protocol for a stepped wedge cluster randomised controlled trial with nested economic and process evaluation of a training package for therapists

Polly-Anna Ashford,[1,*] Clare Knight,[2,*] Margaret Heslin,[3] Allan Clark,[1] Mona Kanaan,[4] Ushma Patel,[5] Freya Stuart,[2] Thomas Kabir,[6] Nick Grey,[7] Hannah Murray,[8] Joanne Hodgekins,[9] Nesta Reeve,[10] Nicola Marshall,[5] Michelle Painter,[10] James Clarke,[5] Debra A Russo,[2] Jan Stochl,[2] Maria Leathersich,[1] Martin Pond,[1] David Fowler,[11] Paul French,[12] Ann Marie Swart,[1] Mary Dixon-Woods,[13] Sarah Byford,[3] Peter B Jones[2,□] and Jesus Perez[5,□]

***Joint first authors**

□ Joint senior authors

1. Norwich Clinical Trials Unit, University of East Anglia, Norwich, UK.
2. Department of Psychiatry, University of Cambridge, Cambridge, UK.
3. King's College London, London, UK.
4. Department of Health Sciences, University of York, York, UK.
5. Cambridgeshire and Peterborough NHS Foundation Trust, Cambridge, UK.
6. The McPin Foundation, London, UK.
7. Sussex Partnership NHS Foundation Trust, Brighton, UK.
8. Oxford University, Oxford, UK.
9. Norwich Medical School, University of East Anglia, Norwich, UK.
10. Norfolk and Suffolk NHS Foundation Trust, Norwich, UK.
11. University of Sussex, Brighton, UK.
12. Manchester Metropolitan University, Manchester, UK
13. THIS Institute, University of Cambridge, Cambridge, UK.

Corresponding author: Jesus Perez

CAMEO Early Intervention Service, Chesterton Medical Centre, 35 Union Lane, Cambridge, CB41PX, Cambridge
Emails: jesus.perez@cpft.nhs.uk; jp440@cam.ac.uk
Phone: +44(0)1223458973

For peer review only

Abstract

Introduction

At least 1 in 4 people treated by the primary care Improving Access to Psychological Therapies (IAPT) programme in England experiences distressing psychotic experiences (PE) in addition to common mental disorder (CMD). These individuals are less likely to achieve recovery. IAPT services do not routinely screen for nor offer specific treatments for CMD including PE. The Tailoring evidence-based psychological therapY for People with common mental disorder including Psychotic EXperiences (TYPPEX) study will evaluate the clinical and cost-effectiveness of an enhanced training for cognitive behavioural therapists that aims to address this clinical gap.

Methods and analysis

This is a multisite, stepped-wedge cluster randomised controlled trial. The setting will be IAPT services within three mental health trusts. The participants will be (i) 56-80 qualified IAPT cognitive behavioural therapists and (ii) 600 service users who are triaged as appropriate for CBT in an IAPT service *and* have psychotic experiences according to the Community Assessment of Psychic Experiences - Positive 15-items Scale (CAPE-P15). IAPT therapists will be grouped into 8 study clusters subsequently randomised to the control-intervention sequence. We will obtain pseudonymous clinical outcome data from IAPT clinical records for eligible service users. We will invite service users to complete health economic measures at baseline, 3, 6, 9 and 12-month follow up. The primary outcome will be the proportion of patients with CMD-PE who have recovered by the end of treatment as measured by the official IAPT measure for recovery.

Ethics and dissemination

The study received the following approvals: South Central – Berkshire Research Ethics Committee on 28/04/2020 (REC reference 20/SC/0135) and HRA on 23/06/2020. An amendment was approved by the Ethics Committee on 01/10/2020 and HRA on 27/10/2020. Results will be made available to patients and the public, the funders, stakeholders in the IAPT services and other researchers.

Trial registration number

ISRCTN93895792

Keywords: Anxiety, depression, cognitive behavioural therapy, common mental disorders, IAPT, primary care, psychosis, psychotic experiences.

For peer review only

Strengths and limitations of this study

- The TYPPEX study will provide the first multisite, stepped-wedge cluster randomised controlled trial to investigate the clinical and cost-effectiveness of an intervention in the UK Improving Access to Psychological Therapies (IAPT) programme.
- The study intervention seeks to address the currently underserved needs of an important group of people accessing IAPT services.
- The stepped-wedge cluster design allows the intervention to be rolled out over time to all potential participants, while reducing operational and logistical challenges.
- The primary limitation is potential loss to follow-up, resulting in missing data for the secondary health economics outcome. This would challenge the internal validity of the conclusions drawn from the study.

Introduction

Psychotic experiences (PE), such as attenuated and/or fragmentary paranoid beliefs and hallucinations, are relatively common in the general population, especially amongst young people.[1] Though systematic reviews and evidence synthesis indicate that 30–40% of those with intense and frequent attenuated psychotic symptoms will transition to a psychotic disorder,[2, 3] studies including individuals with shorter and less intense mental symptoms have shown that far fewer (~10%) make such a conversion.[4, 5] Nonetheless, PE still predict propensity to seek treatment from mental health services[6] and are a marker for severity of other, non-psychotic common mental disorders (CMD), particularly depression and anxiety.[4, 7, 8]

In England, the Improving Access to Psychological Therapies (IAPT) programme is the main provision for people with common mental disorders. Offering UK National Institute for Health and Care Excellence (NICE)-approved psychological therapies, the programme is predominantly based on cognitive behavioural therapy (CBT). National standards specify that 50% of IAPT service users should achieve recovery, a key performance indicator defined by reduction in scores on indices of depression and anxiety. For the first time since its inception in 2008, IAPT met this target in 2016-17, with 51% of people nationally being considered recovered by the end of treatment.[9]

Evidence suggests that at least one in four people treated by IAPT may have a common mental disorder that includes psychotic experiences (CMD-PE). These individuals are at increased risk of not demonstrating recovery.[5, 10] Currently, IAPT neither screens for, nor offers specific treatments for CMD-PE. Treatment protocols focus exclusively on mood disturbance, leaving PE undetected and untreated. Given the complexity and co-morbidity of CMD-PE, standard treatment interventions are likely to be sub-optimal.

Though some effective psychological treatments for PE exist, such as CBT for at-risk mental states,[11, 12] they are dispersed throughout various service settings, and as such are

For peer review only

inadequate to assess or treat the whole condition of CMD-PE. An innovative National Institute for Health Research (NIHR)-funded programme – Tailoring evidence-based psychological therapY for People with common mental disorder including Psychotic EXperiences (TYPPEX) – is targeting this clinical gap. It seeks to adapt and assemble available treatment options for CMD-PE into a practical therapeutic toolbox for cognitive behavioural therapists in IAPT known as CBT Tailoring for Severity (CBT-ts), rolled out via an enhanced training package and supervision programme.

A single-arm, three-site feasibility study (NHS REC reference 19/SC/0077) demonstrated the successful delivery of CBT-ts to 31 therapists and outcome data collection for 153 CAPE+ patients on their caseload, and provided qualitative information used to refine the training intervention (study report available by request from the authors).

This protocol paper describes the TYPPEX stepped wedge cluster Randomised Controlled Trial (TYPPEX swcRCT), which aims to determine the clinical and cost-effectiveness of CBT-ts versus IAPT standard care (SC) in IAPT service users with CMD and also PE, as determined by the Community Assessment of Psychic Experiences - Positive 15-items Scale (CAPE-P15)[13]. This pragmatic trial will be the first to identify and treat people with CMD-PE in IAPT services. It will provide valuable evidence for the development of interventions for this important and underserved population, while adding to the body of knowledge about design and conduct of randomised controlled trials in the IAPT setting.

Methods

This protocol is reported in accordance with Standard Protocol Items: Recommendations for Interventional Trials (SPIRIT) guidance[14] and refers to study protocol version 2.0 dated 4th of August 2020, which was the first version to be implemented. Amendments made to the protocol since commencement of the trial are shown in Table 1. Participant timelines and measures are summarised in Table 2.

-- Table 1 about here –

-- Table 2 about here –

Public and Patient Involvement

An experienced public and patient involvement (PPI) representative contributed significantly to the development of the PPI strategy set out in the grant application and continues to have study oversight as a member of the steering committee. The submission was endorsed by a co-applicant with relevant lived and academic experience and a PPI coordinator with lived experience was employed to facilitate PPI activity.

For peer review only

At the planning stage a Lived Experience Advisory Panel ('LEAP') was convened comprising 11 people with lived experience including diverse community members. The LEAP influenced some research questions extending the scope of a systematic review within the research programme [15] and informing qualitative interview questions.

Since TYPPEX is a pragmatic trial, the study design is largely dictated according to the way that IAPT services operate. The primary outcome measure is a national metric of 'recovery' derived from routinely collected IAPT data, and is not open to alteration. LEAP members positively influenced secondary outcome measures including introducing the CAPE-P15 at final follow up.

LEAP involvement in the development of the intervention (CBT-ts) was highly influential, for example in review of the training resources and ideas about language and delivery. All patient-facing resources are produced in partnership with the LEAP, who have been invaluable in raising issues around acceptability and accessibility. The panel reviewed the feasibility study protocol and gave feedback on adaptations to remote working for the main trial. All ethical submissions undergo thorough PPI review with strong focus on acceptability of consent processes.

PPI representation from IAPT services was not initially possible since primary care services do not routinely screen for PE. However, after the trial's feasibility study, we have expanded involvement to include three people recruited for such study, who have lived experience that matches our target population. We have also expanded involvement to include four IAPT CBT therapists, also drawn from the feasibility study, to ensure acceptability and usability of CBT-ts and research methods within an IAPT setting, together contributing an additional seven perspectives.

People with lived experience will be involved in qualitative data interpretation to provide context and sense-checking of themes and anonymised service-user quotes as part of the process evaluation. LEAP members have co-authored papers and presented posters at public events and dissemination activity will continue to be informed and enhanced by people with lived experience.

PPI will remain reflective and responsive to new insights and the needs of the study, working to the NIHR INVOLVE's Standards for Public Involvement.[16]

Study Setting

The IAPT programme in England began in 2008 with the aim of improving access to evidence-based psychological treatment for CMD, particularly depression and anxiety. It sought to increase public access to National Institute for Health and Care Excellence (NICE)-approved psychological therapies, predominantly cognitive behavioural therapy (CBT), by offering flexible referral routes, including self-referral. The programme has continued to expand over time and currently assesses over 1.3 million people with CMD annually, delivering therapy to approximately 550,000.[9] NHS England has committed to further expansion of the programme, aiming to see 1.9 million patients annually by 2024.[17].

For peer review only

The programme provides treatment for CMD for people aged 17 or over. Its pathways involve a stepped care model, providing steps 2 and 3 of the National Institute for Clinical Excellence (NICE) five-step approach to the treatment of CMD. At step 2 in the IAPT programme, large numbers of service users, with less severe conditions may be allocated to a single therapist. These service users may receive a variety of interventions, including brief face-to-face therapy, telephone support, computerised CBT or guided self-help. At step 3, highly trained CBT therapists manage smaller caseloads of people with more severe CMD. These therapists use more intensive treatment methods, delivering up to 20 individual face-to-face psychological therapy sessions. This clinical setting is immediately below psychiatric treatment by secondary mental health services (NICE step 4.

Step 3 IAPT CBT therapists will have completed a one-year postgraduate diploma (PG Dip) in CBT accredited by the British Association for Behavioural and Cognitive Psychotherapies (BABCP). Entry requirements for the PG Dip include a secured work placement for clinical practice and a BABCP recognised core profession, such as mental health nursing or counselling psychology.[18]

Trial design and Participants

The TYPPEX study is a pragmatic, multi-centre stepped wedge cluster Randomised Controlled Trial (swcRCT) with nested health economic and process evaluations. It involves eight NICE step-3 IAPT teams (clusters) of CBT therapists across three mental health trusts in England: Cambridgeshire and Peterborough NHS Foundation Trust (CPFT), Norfolk and Suffolk NHS Foundation Trust (NSFT) and Sussex Partnership NHS Foundation Trust (SPFT). Each trust (organisational unit within the National Health Services of England and Wales) serves a mixed urban and rural population with a wide socio-economic range, including some high levels of deprivation, covering a total population of approximately 2.8 million.

Clusters will be randomised to different sequences rather than, strictly speaking, different arms. The sequences dictate the order in which each cluster switches from the control to intervention condition. TYPPEX swcRCT has five measurement periods and four steps, with two clusters switching to the intervention condition at each step. As the intervention is cluster randomised at the level of IAPT team, and the delivery of therapy by these teams may be subject to regional variation, each cluster will act as its own internal control. All clusters will start in the control condition and move to the intervention condition (see Figure 1).

The primary outcome of this study will be the proportion of eligible individuals with CMD-PE, as determined by the CAPE-P15 cut-off of 1.47[19], who have recovered by the end of treatment, measured via pseudonymous clinical outcome data obtained from IAPT clinical records and IAPT-defined metrics of recovery, as specified in Table 2.

-- Figure 1 about here –

For peer review only

Eligibility criteria

IAPT therapist inclusion criteria are: (1) qualified IAPT CBT therapist, and (2) willing and able to provide informed consent to receive CBT-ts training and supervision. IAPT therapist exclusion criteria are: (1) does not deliver CBT therapy in current role, (2) works across more than one locality IAPT team (to avoid potential contamination e.g. via team meetings, shared office space), and (3) participated in an earlier TYPPEX feasibility study.

Service user participants are those individuals accepted onto the NICE step-3 IAPT caseload for participating therapists and meeting the study eligibility criteria, which are: (1) accepted onto the IAPT caseload for therapy and therefore meet service-specific inclusion criteria to access IAPT treatment (irrespective of whether service has been accessed previously or not), (2) meet current criteria for IAPT CBT treatment, and (3) assessed for PE, according to the CAPE-P15 questionnaire in their clinical record. Additional inclusion criteria for health economics sub-study only: (4) presence of PE according to the CAPE-P15 score, (5) in the judgement of the treating therapist has sufficient proficiency in English to complete research questionnaires, and (6) able and willing to provide written informed consent.

IAPT service user exclusion criteria are: (1) presence of mental disorder, such as complex and severe depression, based on standard IAPT assessment meriting routine referral to NICE step 4treatment, i.e. to secondary mental health services.

Participant withdrawal

The right of any participant – therapist or service user – to discontinue participation without giving any reason will be respected. People will remain free to withdraw from the trial at any point without prejudicing their further treatment or, for therapists, employment. Reason for withdrawal, if given, will be recorded. Service users who have refused or withdrawn consent for data sharing will be excluded from pseudonymous routine data collection.

Randomisation

IAPT therapists from local teams within participating sites will be recruited and grouped as study clusters before randomisation (without stratification by cluster characteristics) to the control-intervention sequences via computer written code. In most cases, clusters will be formed from a single IAPT team. Some teams may have fewer IAPT CBT therapists available for training due to empty therapist posts or service demands. In these cases, two local IAPT teams will be merged to create a cluster. Once clusters are allocated, IAPT service managers will be approached, and CBT-ts training courses arranged in line with each cluster randomisation point.

Blinding

The study will be conducted in a controlled open format with unblinded assessment; neither the research team nor the therapists that form the study clusters can be blind to the

For peer review only

intervention. IAPT service users will be made aware of the study via an information leaflet given during screening but will not know whether their therapist has received CBT-ts training.

Sample size

CBT-ts training will be delivered to step-3 CBT therapists in eight clusters, randomised at four steps. In each cluster, approximately 7-10 therapists will receive training, with the intention for at least five therapists to deliver therapy throughout each cluster-period, to allow for inevitable therapist turnover and sickness.

Based on our prevalence studies of CMD-PE in IAPT [5, 10] and considering average caseloads of 15-20 services users per therapist over six months, each therapist will deliver therapy to a minimum of three people. Pragmatically, the total number of eligible service users receiving therapy from a participating therapist during the whole trial may be higher, but only control service users who complete therapy during their cluster's control phase, and intervention service users who complete therapy prior to the end of the intervention phase, will be included in the primary outcome analysis. Based on results from our previous TYPPEX feasibility work, we expect that 6-month steps would be sufficient for this.

All service users will be identified as having PE with the CAPE-P15 score threshold of 1.47[19] for both frequency of and distress associated to psychotic experiences at baseline, resulting in a minimum of 15 service users per cluster period. Eight clusters and four randomisation steps result in 20 control condition cluster-periods and 20 intervention condition cluster-periods. 300 IAPT service users will be recruited during the control condition and 300 during the intervention condition across all sites. In addition to the number of clusters and the steps presented above, we have assumed: an intra-cluster correlation coefficient of 0.05, a significance level of 0.05, and an improvement in recovery from 0.39 (control) to 0.58 (intervention) [5, 10]. Based on these assumptions, the proposed sample size will allow us to detect such a difference with just over 80% power. Although this ignores that clustering also happens at the therapist level, it has been shown that ignoring an additional, lower level of clustering is a conservative estimate of the power. This is not sensitive to the choice of intra-cluster correlation coefficient [20].

Also during the feasibility work we found that approximately 50% of the sample providing pseudonymised routine data would consent to complete the health economic measures. Thus, we aim that 150 service users will be consented to complete health economic measures during the control condition and 150 during the intervention phase across all sites.

Procedures

All IAPT service users who begin a course of CBT from the outset of the trial will be asked to complete a CAPE-P15 screening questionnaire and will receive a leaflet during their first treatment session. The leaflet explains that the trial is taking place in the service, and if eligible, their pseudonymous clinical data will be used for the research. Awareness of the

For peer review only

trial is also raised in participating sites using posters. Both the poster and leaflet provide service users with information on the local policy for how they can opt out should they wish their data not to be used for research purposes. The CAPE-P15 screening questionnaire is currently accessible for clinical information as part of routine service use for participating sites.

IAPT service users with PE according to the CAPE-P15 will be asked by their therapist to provide consent to be contacted about participating in the health economic data collection, since this data cannot be collected from clinical records. If consent is obtained, the service user will be sent a participant information sheet and an electronic consent form by email (or paper versions by post) prior to the following treatment session, followed by baseline health economics measures.

Recruited service users will receive standard IAPT care from a participating therapist (control condition) or standard IAPT care from a participating therapist following CBT-ts training (intervention condition). They will be contacted by email or post for follow-up health economics data collection at 3, 6, 9, and 12 months after baseline.

Intervention

CBT-Tailoring for severity (CBT-ts)

CBT-ts is a team-level service improvement intervention. It aims to enhance existing CBT skills of NICE step-3 IAPT CBT therapists and is not a novel therapeutic intervention. Therefore, it is important to highlight that all IAPT service-users receiving CBT under the care of a CBT-ts trained therapist, including those who do not have CMD-PE, may be exposed to the effects of the training.

CBT-ts is based on the CBT framework currently used nationally in IAPT but includes adaptations to existing interventions to specifically address PE. The training takes a knowledge, skills and attitude focus enhancing existing knowledge of CBT. It includes three modules delivered online as workshops supplemented by a blended learning package with access to an online network and provision of written resources. Therapists are provided with a single pack of editable templates, which they may or may not use in their clinical practice.

The structured training programme includes:

Module 1:

- Introduction to the concept of CMD-PE and the relationship between CMD and PE.
- Prevalence and impact in IAPT services.
- Identification of PE.
- Normalisation and validation of CMD-PE.

Module 2:

For peer review only

- Incorporating assessing PE into existing assessments within CBT.
- Suitability for IAPT.
- Understanding CMD-PE maintenance cycles.
- Approaches to the therapeutic conceptualisation and formulation of CMD-PE including co-morbidity.

Module 3:

- Adaptations to existing cognitive behavioural strategies and the application of these to promote the therapeutic change, using change methodologies, such as metaphors, exposure, cognitive restructuring and behavioural experiments.
- Relapse prevention.

Training is supported by six CBT-ts supervision sessions, which commence one month post training and are subsequently delivered monthly, either online or face-to-face. Following completion of training, CBT therapists will identify people with CMD-PE according to the CAPE-P15, and treat them in line with current CBT interventions, incorporating their enhanced skills, as necessary. Treatment duration is non-prescriptive and expected to range between 8 to 14 sessions dependent upon therapists' clinical decisions. Treatment duration is not expected to exceed 20 weekly sessions, in line with current standard guidance in IAPT services, nationally.

Trial data collection on therapist participants will include training attendance, reasons for non-attendance, and implementation of the resources and components of the CBT-ts 'toolkit' with therapists' CAPE+ caseload during the supervision period. Therapists' will be assessed on their overall adherence and engagement with CBT-ts.

Standard care

The control comparator is standard CBT care delivered within IAPT services prior to CBT-ts training. CBT is delivered as part of range of interventions offered at step 3 within the NICE stepped care model. CBT treatment protocols are delivered in accordance with NICE guidance for people with CMD. Treatment duration varies according to presenting problem and minimum dose necessary to achieve recovery, but NICE recommends individuals should receive up to 20 weekly sessions unless they recover beforehand (https://www.nice.org.uk/guidance/cg123/chapter/1-guidance).

Outcomes

A summary of study outcomes is shown in Table 3, and schedules of outcome measures for service users and therapists are provided in Tables 2 and 4.

For peer review only

-- Table 3 about here –

-- Table 4 about here –

Process evaluation

The aim of the process evaluation is to assess the views of all stakeholders involved in the experience and delivery of the TYPPEX swcRCT and CBT-ts, and to investigate the implementation of CBT-ts as designed, including any influences on uptake, delivery, and fidelity. An overview of the key themes explored with each stakeholder group is given in Table 5.

-- Table 5 about here –

The aim of sampling for the process evaluation will be to achieve theoretical saturation [21], where further new data is unlikely to achieve further insights or to add to analytic depth. Up to 32 service users across the eight clusters with scores of 1.47 and above on the CAPE-P15 and who have completed at least two CBT treatment sessions with a CBT-ts trained therapist will be recruited including those who have completed treatment, those who are still undergoing therapy and those who dropped out of treatment. Where possible, purposive sampling will be used to ensure diversity of gender and ethnicity and exposure to number of sessions of therapy.

Between 16 and 24 IAPT therapists (with a minimum of two per cluster) who completed CBT-ts training and subsequently delivered at least three treatment sessions to a minimum of two service-users scoring 1.47 and above on the CAPE-P15 will be recruited. Additionally, we will conduct 18-25 interviews with IAPT clinical supervisors/managers from participating IAPT teams who line manage or supervise a CBT-ts trained therapist, senior non-clinical NHS managers with responsibility for delivery of IAPT services at Trust level or responsibility for commissioning IAPT services, and members of the trial team and collaborators who contributed to the design, development, or implementation of TYPPEX. In all cases, purposive sampling will be used to ensure representation of a wide range of perspectives and experiences.

Data management

Pseudonymised outcome data will be received by Norwich Clinical Trials Unit from sites and uploaded to a central study database stored on servers based at the University of East Anglia. Health economic questionnaire data will be collected using REDCap data collection software (https://www.project-redcap.org). Questionnaires will be completed online or by post by service users. Pseudonymised clinical data will be linked to consented participants' health economics data held by NCTU.

For peer review only - http://bmjopen.bmj.com/site/about/guidelines.xhtml

Data collection will include retrospectively recorded IAPT data from 23rd March 2020 until trial start. This data, covering the period of UK lockdown during the COVID-19 pandemic, will provide information on the nature of IAPT service provision during this time, and any changes in baseline morbidity. The aim of the data collection is to provide insight into standard of care and baseline severity during the pandemic, to assist decision-making by the trial oversight committees.

Data will be handled in accordance with a data management plan and comply with the principles of the International Conference on Harmonisation Good Clinical Practice, within the Standard Operating Procedures for Data Management in NCTU and where appropriate with UEA IT procedures. Interview transcriptions will be anonymised in adherence with the "Guidance on Anonymisation" issued by the UK Data Service (https://www.ukdataservice.ac.uk/manage-data/legal-ethical/anonymisation.aspx). Anonymised data will be stored in password-protected files on the firewall-protected University of Cambridge servers.

Statistical analyses

The primary analysis will compare IAPT-defined recovery of service users with CMD-PE as determined by the CAPE-P15 cut-off of 1.47 in the control phase with those in the intervention phase (post-training). Recovery proportions and means will be compared at individual therapist and team level after adjusting for the stepped-wedge cluster design. Adjustment will also be made for the baseline characteristics and other covariates. Time will be included as a set of indicator variables.

Analyses of clinical effectiveness will be based on the Intention to Treat (ITT) population with all available follow-up data from all new service users with CMD-PE on the caseload of participating therapists, controlling for baseline scores on depression and anxiety disorder specific measures. The primary ITT analysis is intended to provide inferences regarding the effectiveness of the intervention overall. It does not provide inferences regarding the causal effect of the intervention itself, but on the intervention as deployed in 'real life', therefore compliance information is not necessary to ensure that the ITT analysis is valid. As soon as a therapist has their first session of CBT-ts training, they begin to practise differently, so this date will mark the beginning of their intervention phase.

A logistic mixed effects regression model will be used for the primary outcome analysis. The cluster effect by both the therapist team and therapist will be considered in the multilevel analysis. Adjustment for potential prognostic variables will be agreed prior to analysis between the trial statisticians and the Chief Investigator.

Intra-cluster correlation coefficients and 95% confidence intervals will be reported for the primary and secondary outcomes. All analyses will be two-tailed and at the 5% level of significance.

For peer review only

Summary statistics of baseline characteristics at the cluster and the individual level will be reported where appropriate. Analyses will be conducted in Stata or R. Publication of results will include a participants/cluster flow diagram, and results will be reported according to the CONSORT statement for stepped wedge designs.

Economic analysis

The economic evaluation will be conducted covering the period from baseline to 12-month follow-up and will take the NHS/Personal Social Services perspective preferred by NICE[22]. The intervention will be costed taking a bottom-up (micro-costing) approach[23] using data collected on face-to-face contacts from IAPT records and accounting for the ratio of direct face-to-face to indirect non-face-to-face time. Data on indirect time, including preparation and supervision, will be collected directly from the therapists. Details of resources required to deliver the CBT-ts training and supervision will be provided by the TYPPEX research team. Use of all other health and social care services will be collected via a modified version of the Adult Service Use Schedule for Early Intervention (EI-ADSUS; Table 1) collected using REDCap data collection software at baseline (covering the last 3 months) and 3-, 6-, 9-, and 12-months (covering the period since the last interview). Resource use data will be combined with nationally applicable unit costs.[24, 25] Productivity losses (applicable to sensitivity analyses only) will be calculated using the human capital approach by multiplying days off work for mental health reasons by the national average wage rate.[26]

Our primary economic analysis will be a cost-utility analysis using Quality Adjusted Life Years (QALYs) derived from the EuroQol measure of health-related quality of life (EQ-5D) based on complete case data. The EQ-5D-5L has been proposed as being more sensitive to change in mental health populations compared to the EQ-5D-3L.[27] However, there is little evidence to support this hypothesis. Therefore, we will compare the psychometric properties of the 3L and 5L versions. The version of the EQ-5D used in this analysis will be dependent on analyses comparing the two. QALYs will be calculated by applying appropriate utility weights to EQ-5D health states[28] and using the total area under the curve approach with linear interpolation between assessment points.[29] A secondary analysis will additionally explore cost-effectiveness using the primary outcome measure: IAPT-defined recovery.

Costs and outcomes will be compared at the final follow-up point and presented as mean values by trial phase with standard deviations. Mean differences in costs and 95% confidence intervals will be obtained by nonparametric bootstrap regressions to account for the non-normal distribution commonly found in economic data[30]. Incremental cost-effectiveness ratios (ICERs) will be calculated. Uncertainty will be explored using cost-effectiveness planes and cost-effectiveness acceptability curves (CEACs) based on the net-benefit approach.[31] These curves are an alternative to confidence intervals around incremental cost-effectiveness ratios and show the probability that one intervention is cost effective compared to the other for a range of values that a decision maker would be willing to pay for an additional unit of an outcome. To provide more relevant treatment-effect estimates, all economic analyses will include adjustment for stepped-wedge cluster design, relevant baseline characteristics and other covariates[32], in line with the clinical analyses.

For peer review only

Sensitivity analyses will be conducted to explore the impact of missing data (using multiple imputation) and to explore cost-effectiveness from a broader perspective, including productivity losses because of time off work due to illness. Additionally, to assess the impact of missing data, we will compare those with economic data to the full sample included in the main clinical analysis to examine any potential biases in terms of demographic and clinical factors.

Process evaluation analysis

Analysis of process evaluation data will be based on the constant comparative method.[33,34] Practically, this will be achieved through multiple levels of analysis, systematically comparing data on different questions and from different sources (quantitative and qualitative) to achieve thematically ordered synthesis. Data analysis will take place after each stage of data collection so the analysis from each stage can inform data collection in the next. Some data analysis will also take place in parallel. The data will initially be subject to open coding using a combination of pre-selected questions and sensitising constructs identified from the literature. Codes will be increasingly grouped into higher-order explanatory categories through comparison and refinement in rounds of discussion and sense-making. A set of overarching thematic categories will be agreed and used as a framework for further, more deductive, coding across the whole data set.

Trial Status

The trial was opened to IAPT service user recruitment in March 2021, with the first randomisation point scheduled for September 2021. Recruitment will continue until September 2023. The final 12 month follow-up is expected to be completed by October 2024.

Ethics and dissemination

This study was approved by the South Central – Berkshire Research Ethics Committee (REC reference 20/SC/0135 on 28/04/2020 and HRA on 23/06/2020). Subsequent amendments are shown in Table 1.

The study is jointly sponsored by the University of Cambridge and Cambridgeshire and Peterborough NHS Foundation Trust. It was adopted onto the NIHR trial portfolio on 16/04/2020.

CBT-ts, including therapist training and supervision, is low risk for all participants. IAPT service users scoring above the CAPE-P15 thresholds for PE on whom data is being pseudonymously collected are under the care of the IAPT service for the duration of their participation and are subject to normal IAPT safety procedures. No specific risks, untoward incidents or adverse events related to CBT-ts are anticipated as the intervention aims to up-skill therapists rather than offering a new therapeutic intervention. This approach was supported by the results of a feasibility study as part of the wider TYPPEX research programme, during which no related serious adverse events were reported.

For peer review only

Safety outcome variables will be collected retrospectively each month from the routinely collected pseudonymous data. Aggregated safety information will be reported to the TYPPEX Data Monitoring and Ethics Committee (DMEC) throughout the randomisation condition. In addition, Principal Investigators, IAPT managers and therapists will be requested to report complaints relating to TYPPEX by eligible participants to the trial team.

The clinical data generated during therapy (CAPE-P15, GAD-7, PHQ-9 and ADSM) is recorded as part of routine care and is used by this trial via an opt-out consent process. This is designed to remove the burden on IAPT therapists with respect to recruitment, and to ensure a representative sample.

A sub-group of service users are consented via an opt-in process to provide additional data outside of the clinic setting. This includes cost and quality of life health economic data, and follow-up clinical data at 12 months (see Table 3). This data will be linked with their IAPT clinical record.

Results from this trial will be presented at national and international conferences, including the Schizophrenia International Research Society (SIRS) Conference and IEPA *Early Intervention in Mental Health* Conference. Dissemination will also occur through the submission of a primary article on the outcomes and several subsequent articles considering the effects of the intervention stratified by service user and therapist variables. Primary outcomes will be disseminated more widely as part of a theory-driven implementation plan, developing a community of practise (CoP) including patients, the public, economists and systems engineers experienced in care pathway design and evaluation, in addition to stakeholders in the IAPT services.

The training materials will be shared electronically and will be widely available to the NHS.

Contributors

PBJ and JP are co-CIs for and designed this trial. P-AA is the trial manager. CK was manager for the wider research programme. UP led the design and development of the intervention, supported by DAR. JH, NR, NM, JC, MP, NG, HM, DF and PF advised on the development of the intervention. TK leads on the PPI strategy. FS supports all elements of the PPI strategy and, with DAR, also delivers research assistant functions for the trial. AC and MK contributed to the trial design and oversee the statistical analysis. JS advises on statistical matters. MH and SB lead the health economic component of the trial. MD-W oversees qualitative analyses and process evaluation. ML and MP are responsible for trial data management. AMS oversees all operations at the Clinical Trials Unit. P-AA, CK, MH, AC, MK and JP wrote the first draft of this manuscript. All authors reviewed, contributed to, and commented on the final draft.

Funding

For peer review only

This paper presents independent research funded by the National Institute for Health Research (NIHR) under its Programme Grants for Applied Research Programme (Reference Number RP-PG-0616-20003). The views expressed are those of the author(s) and not necessarily those of the NHS, the NIHR or the Department of Health.

The funding body had no role in study design; collection, analysis, or interpretation of data; the writing of the report; or decision to submit the paper. The first author had full access to all data and had final responsibility for the decision to submit for publication.

Competing interests

None declared.

References

1. Linscott RJ, van Os J. An updated and conservative systematic review and meta-analysis of epidemiological evidence on psychotic experiences in children and adults: on the pathway from proneness to persistence to dimensional expression across mental disorders. *Psychol Med* 2013;43(6):1133-49 doi:10.1017/S0033291712001626.

2. Fusar-Poli P, Bonoldi I, Yung AR, Borgwardt S, et al. Predicting psychosis: meta-analysis of transition outcomes in individuals at high clinical risk. *Arch Gen Psychiatry* 2012;69(3):220-9 doi:10.1001/archgenpsychiatry.2011.1472.

3. Addington J, Epstein I, Liu L, et al. A randomized controlled trial of cognitive behavioral therapy for individuals at clinical high risk of psychosis. *Schizophr Res* 2011;125(1):54-61 doi:10.1016/j.schres.2010.10.015.

4. Hui, C, Morcillo, C, Russo, DA, et al. Psychiatric morbidity, functioning and quality of life in young people at clinical high risk for psychosis. *Schizophr Res* 2013;148(1–3):175–180.

5. Perez J, Russo DA, Stochl J, et al. Common mental disorder including psychotic experiences: trailblazing a new recovery pathway within the Improving Access to Psychological Therapies programme in England. *Early Interv Psychia* 2017;12(3):497–504 doi:https://doi.org/10.1111/eip.12434.

6. Devylder JE, Oh HY, Corcoran CM, et al. Treatment seeking an unmet need for care among persons reporting psychosis-like experiences. *Psychiatr Serv* 2014;65(6):774–780 doi:https://doi.org/10.1176/appi.ps.201300254.

7. Wigman JT, Nierop M.V, Vollebergh WA, et al. Evidence that psychotic symptoms are prevalent in disorders of anxiety and depression, impacting on illness onset, risk, and severity–implications for diagnosis and ultra-high risk research. *Schizophr Bull* 2012;38(2):247–257 doi:https://doi.org/10.1093/schbul/sbr196.

8. Stochl J, Khandaker GM, Lewis G, et al. Mood, anxiety and psychotic phenomena measure a common psychopathological factor. *Psychol Med* 2015;45(7):1–11. doi:10.1017/S003329171400261X.

9. NHS Digital. Psychological Therapies, Annual report on the use of IAPT services - England, 2017-18 (online). https://digital.nhs.uk/data-and-information/publications/statistical/psychological-

For peer review only

therapies-annual-reports-on-the-use-of-iapt-services/annual-report-2017---18 (accessed September 2020).10. Knight C, Russo D, Stochl J, et al. Prevalence of and recovery from common mental disorder including psychotic experiences in the UK Primary Care Improving Access to Psychological Therapies (IAPT) Programme. *J Affect Disord* 2020;(1):272:84-90 doi:10.1016/j.jad.2020.04.015.

11. Morrison AP, French P, Stewart SL, Birchwood M, Fowler D, Gumley AI, Jones PB, Bentall RP, Lewis SW, Murray GK, Patterson P, Brunet K, Conroy J, Parker S, Reilly T, Byrne R, Davies LM, Dunn G. Early detection and intervention evaluation for people at risk of psychosis: multisite randomised controlled trial. *BMJ* 2012;(5)344:e2233.

12. van der Gaag M, Nieman DH, Rietdijk J, Dragt S, Ising HK, Klaassen RM, Koeter M, Cuijpers P, Wunderink L, Linszen DH. Cognitive behavioral therapy for subjects at ultrahigh risk for developing psychosis: a randomized controlled clinical trial. *Schizophr Bull* 2012;38(6):1180-8.

13. Capra C, Kavanagh DJ, Hides L, et al. Current CAPE-15: a measure of recent psychotic-like experiences and associated distress. *Early Interv Psychia* 2017;11(5):411–417 doi: https://doi.org/10.1111/eip.12245.

14. Chan A-W, Tetzlaff JM, Altman DG, et al. SPIRIT 2013 Statement: Defining standard protocol items for clinical trials. *Ann Intern Med* 2013;158(3):200-207.

15. Soneson E, Russo D, Stochl J, et al. Psychological interventions for people with psychotic experiences: A systematic review and meta-analysis of controlled and uncontrolled effectiveness and economic studies. *Aust N Z J Psychiatry* 2020;54(7):673-695. doi: 10.1177/0004867420913118.

16. NIHR Involve. National Standards for Public Involvement in Research V1 March 2018 (online). https://www.invo.org.uk/posttypepublication/national-standards-for-public-involvement/ (accessed November 2020).

17. Clark DM. IAPT at 10: achievements and challenges, 2019 (online). https://www.england.nhs.uk/blog/iapt-at-10-achievements-and-challenges/ (accessed September 2019).

18. British Association for Behavioural & Cognitive Psychotherapies (BABCP) training in cognitive behavioural therapy: https://babcp.com/Careers/Training-in-CBT (accessed 22/04/2022)

19. Bukenaite A, Stochl J, Mossaheb N, et al. Usefulness of the CAPE-P15 for detecting people at ultra-high risk for psychosis: Psychometric properties and cut-off values. *Schizophr Res* 2017;189:69-74. doi: 10.1016/j.schres.2017.02.017.

20. Hussey MA, Hughes JP. Design and analysis of stepped wedge cluster randomized trials. *Contemp Clin Trials* 2007;28(2):182-91.

21. Guest G, Namey E, Chen M. A simple method to assess and report thematic saturation in qualitative research. PLoS One. 2020;15(5):e023207622. National Institute for Health and Care Excellence. Guide to the methods of technology appraisal 2013. London: NICE 2013.

23. Drummond MF, Sculpher MJ, Torrance GW, et al. Methods for the economic evaluation of health care programmes. New York: Oxford University Press 2005.

24. Curtis L, Burns A. Unit Costs of Health and Social Care 2018. University of Kent, Canterbury: Personal Social Services Research Unit 2018.

25. NHS improvement. 2017/18 Reference Costs (online). https://webarchive.nationalarchives.gov.uk/20200501111106/https://improvement.nhs.uk/documents/6468/201718_reference_costs_data_and_guidance.zip/ (accessed March 2020).

26. Koopmanschap MA, Rutten FF. A practical guide for calculating indirect costs of disease. *Pharmacoeconomics* 1996 Nov;10(5):460-6. doi:10.2165/00019053-199610050-00003.

27. Camacho EM, Shields G, Lovell K, et al. A (five-) level playing field for mental health conditions?: exploratory analysis of EQ-5D-5L-derived utility values. *Qual Life Res* 2018;27(3):717-24.

28. Dolan P, Gudex C, Kind P, et al. A social tariff for EuroQol: results from a UK general population survey. York: University of York, Centre for Health Economics 1995.

29. Manca A, Hawkins N, Schulpher MJ. Estimating mean QALYs in trial-based cost-effectiveness analysis: the importance of controlling for baseline utility. *Health Econ* 2005;14(5): 487-496.

30. Thompson SG, Barber JA. How should cost data in pragmatic randomised trials be analysed? *BMJ*. 2000;320:1197-200.

31. Briggs AH. A Bayesian approach to stochastic cost-effectiveness analysis. *Health Econ* 1999;8:257-261.

32. Assmann SF, Pocock SJ, Enos LE, et al. Subgroup analysis and other (mis) uses of baseline data in clinical trials. *Lancet*. 2000 Mar 25;355(9209):1064-9.

33. Charmaz K. Constructing grounded theory. London: Sage Publications 2014.

34. Charmaz K. Grounded theory: Objectivist and constructivist methods. In: Denzin NK, Lincoln YS, eds. Strategies for qualitative inquiry (2nd ed.). Thousand Oaks, CA: Sage; 2003:249-291.

35. Kroenke K, Spitzer RL, Williams JB. The PHQ-9: validity of a brief depression severity measure. *J Gen Intern Med* 2001;16(9):606–613. doi:10.1046/j.1525-1497.2001.016009606.x.

36. Spitzer RL, Kroenke K, Williams JB, et al. A brief measure for assessing generalized anxiety disorder: the GAD-7. *Arch Intern Med* 2006;166(10):1092–1097 doi:https://doi.org/10.1001/archinte.166.10.1092.

37. Perez J, Jin H, Russo DA, et al. Clinical and cost-effectiveness of tailored, intensive liaison between primary and secondary care to detect individuals at risk of a first psychotic illness: a theory-based, cluster-randomised controlled trial. *Lancet Psychiatry*. 2015;2(11):984-93.

38. The National Collaborating Centre for Mental Health, 2018. The improving access to psychological therapies manual (online). https://www.rcpsych.ac.uk/docs/default-source/ improving-care/nccmh/the-iapt-manual–final–republished-7-3-18.pdf?sfvrsn= a192d1af_0 (accessed September 2020).

39. Knight C, Russo D, Stochl J, et al. More sensitive identification of psychotic experiences in common mental disorder by primary mental healthcare services - effect on prevalence and recovery: casting the net wider. *BJPsych Open* 2020;6(6):e136.

40. Group TE. EuroQol-a new facility for the measurement of health-related quality of life. *Health Policy* 1990;16(3):199-208.

41. Herdman M, Gudex C, Lloyd A, et al. Development and preliminary testing of the new five-level version of EQ-5D (EQ-5D-5L). *Qual Life Res* 2011;20(10):1727-36.

For peer review only

42. Stochl J, Croudace T, Perez J, et al. Usefulness of EQ-5D for evaluation of health-related quality of life in young adults with first-episode psychosis. *Qual Life Res* 2013;22(5):1055-63.

43. Lovett R, Cooper S, NICE Science policy and research programme. NICE to support new valuation study for England for EQ-5D-5L questionnaire (online). https://www.nice.org.uk/news/blog/nice-to-support-new-valuation-study-for-england-for-eq-5d-5l-questionnaire (accessed May 2021)

For peer review only

Figure 1. Figure 1: Stepped wedge trial diagram

Table 1: Summary of ethical amendments

Protocol	Primary reasons for amendment
2.0, 04/08/2020	• Addition of secondary clinical outcomes (Reliable Recovery and Reliable Improvement).<br>• Addition of retrospective pseudonymous clinical data collection from the beginning of UK lockdown due to COVID-19, until randomisation.<br>• Intervention adaptation: training to be delivered either online or face-to-face.<br>• Changes to the service user consent process: moving from full consent on tablet devices to consent to contact followed by full consent online at home.
3.0, 12/05/2021	• Video conferencing e.g. Zoom included as an option for Process Evaluation interviews.<br>• Removal of restriction of only teams from the same NHS Trust merging to form a cluster.
3.0 (no change to protocol)<br><br>Non-substantial amendment dated 28/06/2021	Approval for three additional data collection forms:<br><br>• 1) To record the training dates on which consented therapists are due to attend their CBT-ts training, actual completion dates, and reasons for non-attendance.<br>• 2) A non-identifiable implementation record to be completed for each service user brought to supervision by each therapist post CBT-ts training, to be completed by the supervisor.<br>• 3) A per-therapist record of adherence and engagement completed by the supervisor during post-training supervision.
4.0,<br><br>16/08/2021	• Addition of secondary outcome to compare changes in CAPE-P15 scores between the beginning and end of therapy. This change includes the collection of CAPE-P15 as part of the IAPT clinical data collection at the final CBT therapy session.
5.0,<br><br>13/12/2021	• Protocol edited to clarify the timing of CAPE-P15 collection: before the end of session 3, or as early as clinically appropriate. It will then be collected again at the penultimate or final session of therapy.<br>• Process evaluation changes: to capture their ‘journey’ through the trial, therapists will be interviewed up to three times following training and during the trial rather than a single interview. Therefore, the eligibility criteria have been modified, removing the requirement to have delivered a minimum of three treatment session to at least two CAPE+ IAPT services users. The total number of therapists required to be interviewed has also been reduced to accommodate this.

For peer review only

Table 3: Summary of study outcomes

Category	Measure	Primary/Secondary	Details
Service User Recovery	IAPT-defined 'Recovery'	Primary	The primary outcome is the proportion of IAPT service-users with CAPE-P15 score of 1.47 or above who have recovered by the end of treatment. Recovery is a national IAPT programme performance metric, with an individual deemed recovered if they scored above the clinical cut-off on the PHQ-9[35] and/or GAD-7[36] before treatment, i.e. 10 and 8 respectively, and below the clinical cut-off at the end of treatment on both measures.[37] If recorded, an anxiety disorder specific measure (ADSM) is used in place of the GAD-7 if that is the focus of treatment.[38]
	IAPT-defined 'Recovery'	Secondary	As for the primary outcome, but for IAPT service-users reaching the lower threshold score for CMD-PE (CAPE-P15 score of 1.30 and above).[39]
	IAPT-defined 'Reliable Improvement'	Secondary	Service users are considered reliably improved if they show any improvement in scores on the appropriate outcome measures between pre and post treatment, that exceeds the measurement error of the scales.[38]
	IAPT-defined 'Reliable Recovery'	Secondary	Service users are considered reliably recovered if they meet both criteria for Reliable Improvement and for Recovery.[38]
Health economic measures	Modified Adult Service Use Schedule for Early Intervention (EI-ADSUS)	Secondary	Individual-level resource use will be measured using a modified version of Adult Service Use Schedule for Early Intervention (EI-ADSUS). This was developed in previous research with similar populations[37] and adapted for electronic participant self-completion. It will include all-cause hospital and community-based health and social care resource use as well as information on time off work due to mental health.
	EuroQol measure of health-related quality of life 3 level (EQ-5D-3L)	Secondary	Health related quality of life will be measured using the EQ-5D-3L, a generic, preference-based measure based on five dimensions (mobility, self-care, usual activities, pain/discomfort, and anxiety/depression). Each dimension is rated on three levels (no problems, some problems, and severe problems).[40]

For peer review only

	EuroQol measure of health-related quality of life 5 level (EQ-5D-5L)	Secondary	The EQ-5D-5L is also a health-related quality of life measure based on five dimensions (mobility, self-care, usual activities, pain/discomfort and anxiety/depression), but each dimension is rated on five levels (no problems, slight problems, moderate problems, severe problems, and extreme problems.[41]<br><br>It has been demonstrated that the EQ-5D-3L is a useful, valid instrument in young people with emerging psychotic symptoms.[42] However, the new five-level version, may prove more sensitive to change and is now recommended for economic evaluation by NICE[43] but has not been validated in this population. Therefore, we will test the sensitivity of the EQ-5D-5L in comparison to the EQ-5D-3L.
Therapist Adherence	Supervision checklist	Secondary	Therapist adherence measured using a supervision checklist and adherence score completed during the 6 monthly supervision sessions held after CBT training. Trial supervisors will review and collate therapy session notes and rate adherence to the CBT-ts approach.

For peer review only

Table 2: Schedule of IAPT user enrolment, interventions, and assessments.

	**STUDY PERIOD (CONTROL AND RANDOMISED CONDITIONS)**						
	Screening	Baseline	Treatment	Follow Up			
**TIMEPOINT**	**-t1***	**-t2**	**Up to 20 sessions**	**3**	**6**	**9**	**12**
**ENROLMENT**							
Eligibility screen	x						
Consent to contact (for health economics measures only)							
Informed consent (for health economics measures only)		x					
IAPT CBT standard care			———				
**ASSESSMENTS**							
PHQ-9**	x	x	X				x
GAD-7/ADSM**	x	x	X				x
EQ-5D-3L		x		x	x	x	x
EQ-5D-5L		x		x	x	x	x
EI-ADSUS		x		x	x	x	x
CAPE P-15	x						x

*The duration between the screening visit and baseline is anticipated to be 1-3 weeks, depending on typical frequency of therapy sessions in IAPT service. During this time the CAPE-P15 is scored and eligibility is confirmed. CAPE+ IAPT users are approached to participate in the health economics data collection during the baseline visit. Clinical data is obtained for all CAPE+ IAPT users unless they opt out.

**PHQ-9 and GAD-7 are routine IAPT clinical measures and are collected during every clinical contact.

For peer review only

Table 4: Schedule of IAPT therapist enrolment, interventions, and assessments.

	Trial set-up	Randomisation	Control condition	Intervention condition
**IDENTIFICATION**				
IAPT teams and potential therapists identified and grouped to form clusters	x			
**ENROLMENT AND RANDOMISATION**				
Informed consent	x			
Clusters randomised		x		
**INTERVENTION**				
CBT-ts training				x
CBT-ts supervision				x
**ASSESSMENTS**				
Adherence and engagement record				x

Table 5: Key themes explored in the process evaluation for each stakeholder group

Stakeholder Group	Key Themes
Service users	• Experiences of treatment and therapist<br>• Views on improving IAPT services<br>• Views on CBT-ts
Therapist:	• Design and goals of CBT-ts<br>• Experience of the CBT-ts training<br>• Experience of delivery and fidelity
Line Managers	• Design and goals of CBT-ts<br>• Experience of the CBT-ts training<br>• Experience of delivery and fidelity at the therapist-service user level<br>• Experience of delivery and fidelity at the IAPT service level
Study Team and collaborators	• Rationale for CBT-ts<br>• Implementation and components<br>• Outcomes and measurement<br>• Modifications and changes to CBT-ts
Senior NHS Managers/Commissioners	• Relevance of CBT-ts in IAPT<br>• Implementation of CBT-ts model<br>• Negotiating change<br>• Measuring success

For peer review only

Cluster		Control	Step 1	Step 2	Step 3	Step 4
1	RANDOMISATION					I
2						I
3					I	I
4					I	I
5				I	I	I
6				I	I	I
7			I	I	I	I
8			I	I	I	I
		Months 1-6	Months 7-12	Months 13-18	Months 19-24	Months 25-30

Key:

Control phase
Intervention phase (I)

For peer review only

BMJ Open

Treating common mental disorder including psychotic experiences in the primary care Improving Access to Psychological Therapies programme (the TYPPEX study): protocol for a stepped wedge cluster randomised controlled trial with nested economic and process evaluation of a training package for therapists

Journal:	*BMJ Open*
Manuscript ID	bmjopen-2021-056355.R2
Article Type:	Protocol
Date Submitted by the Author:	19-May-2022

Complete List of Authors:	Ashford, Polly-Anna; University of East Anglia, Department of Clinical Psychology and Psychological Therapies, Norwich Medical School<br>Knight, Clare; University of Cambridge Department of Psychiatry, Psychiatry<br>Heslin, Margaret; King's College London Institute of Psychiatry Psychology and Neuroscience, King's Health Economics<br>Clark, Allan; University of East Anglia, Norwich Medical School<br>Kanaan, Mona; University of York, Health Sciences<br>Patel, Ushma; Cambridgeshire and Peterborough NHS Foundation Trust<br>Stuart, Freya; University of Cambridge Department of Psychiatry<br>Kabir, Thomas; The McPin Foundation<br>Grey, Nick; Sussex Partnership NHS Foundation Trust; University of Sussex, Psychology<br>Murray, Hannah; University of Oxford,<br>Hodgekins , J ; University of East Anglia, Norwich Medical School<br>Reeve, Nesta; Norfolk and Suffolk NHS Foundation Trust<br>Marshall, Nicola; Cambridgeshire and Peterborough NHS Foundation Trust<br>Painter, Michelle; Norfolk and Suffolk NHS Foundation Trust<br>Clarke, James; Cambridgeshire and Peterborough NHS Foundation Trust<br>Russo, Debra; University of Cambridge Department of Psychiatry<br>Stochl, Jan; University of Cambridge, Department of Psychiatry<br>Leathersich, Maria; University of East Anglia Norwich Medical School<br>Pond, Martin; University of East Anglia Norwich Medical School<br>Fowler, David; University of Sussex,<br>French, Paul; Manchester Metropolitan University<br>Swart, Ann Marie; University of East Anglia, Norwich Medical School<br>Dixon-Woods, Mary ; University of Cambridge Primary Care Unit, THIS Institute (The Healthcare Improvement Studies Institute)<br>Byford, Sarah; King's College London, Centre for the Economics of Mental and Physical Health<br>Jones, Peter; University of Cambridge, Department of Psychiatry<br>Perez, Jesus; Cambridgeshire and Peterborough NHS Foundation Trust; IBSAL, Medicine, Universidad de Salamanca

**Primary Subject Heading**:	Mental health
Secondary Subject Heading:	Health services research, Patient-centred medicine, General practice / Family practice
Keywords:	Anxiety disorders < PSYCHIATRY, Schizophrenia & psychotic disorders < PSYCHIATRY, PRIMARY CARE, Depression & mood disorders < PSYCHIATRY

SCHOLARONE™
Manuscripts

Treating common mental disorder including psychotic experiences in the primary care Improving Access to Psychological Therapies programme (the TYPPEX study): protocol for a stepped wedge cluster randomised controlled trial with nested economic and process evaluation of a training package for therapists

Polly-Anna Ashford,[1,*] Clare Knight,[2,*] Margaret Heslin,[3] Allan Clark,[1] Mona Kanaan,[4] Ushma Patel,[5] Freya Stuart,[2] Thomas Kabir,[6] Nick Grey,[7] Hannah Murray,[8] Joanne Hodgekins,[9] Nesta Reeve,[10] Nicola Marshall,[5] Michelle Painter,[10] James Clarke,[5] Debra A Russo,[2] Jan Stochl,[2] Maria Leathersich,[1] Martin Pond,[1] David Fowler,[11] Paul French,[12] Ann Marie Swart,[1] Mary Dixon-Woods,[13] Sarah Byford,[3] Peter B Jones[2,[illegible]] and Jesus Perez[5,[illegible]]

***Joint first authors**

[illegible] Joint senior authors

1. Norwich Clinical Trials Unit, University of East Anglia, Norwich, UK.
2. Department of Psychiatry, University of Cambridge, Cambridge, UK.
3. King's College London, London, UK.
4. Department of Health Sciences, University of York, York, UK.
5. Cambridgeshire and Peterborough NHS Foundation Trust, Cambridge, UK.
6. The McPin Foundation, London, UK.
7. Sussex Partnership NHS Foundation Trust, Brighton, UK.
8. Oxford University, Oxford, UK.
9. Norwich Medical School, University of East Anglia, Norwich, UK.
10. Norfolk and Suffolk NHS Foundation Trust, Norwich, UK.
11. University of Sussex, Brighton, UK.
12. Manchester Metropolitan University, Manchester, UK
13. THIS Institute, University of Cambridge, Cambridge, UK.

Corresponding author: Jesus Perez

CAMEO Early Intervention Service, Chesterton Medical Centre, 35 Union Lane, Cambridge, CB41PX, Cambridge
Emails: jesus.perez@cpft.nhs.uk; jp440@cam.ac.uk
Phone: +44(0)1223458973

For peer review only

Abstract

Introduction

At least 1 in 4 people treated by the primary care Improving Access to Psychological Therapies (IAPT) programme in England experiences distressing psychotic experiences (PE) in addition to common mental disorder (CMD). These individuals are less likely to achieve recovery. IAPT services do not routinely screen for nor offer specific treatments for CMD including PE. The Tailoring evidence-based psychological therapY for People with common mental disorder including Psychotic EXperiences (TYPPEX) study will evaluate the clinical and cost-effectiveness of an enhanced training for cognitive behavioural therapists that aims to address this clinical gap.

Methods and analysis

This is a multisite, stepped-wedge cluster randomised controlled trial. The setting will be IAPT services within three mental health trusts. The participants will be (i) 56-80 qualified IAPT cognitive behavioural therapists and (ii) 600 service users who are triaged as appropriate for CBT in an IAPT service *and* have psychotic experiences according to the Community Assessment of Psychic Experiences - Positive 15-items Scale (CAPE-P15). IAPT therapists will be grouped into 8 study clusters subsequently randomised to the control-intervention sequence. We will obtain pseudonymous clinical outcome data from IAPT clinical records for eligible service users. We will invite service users to complete health economic measures at baseline, 3, 6, 9 and 12-month follow up. The primary outcome will be the proportion of patients with CMD-PE who have recovered by the end of treatment as measured by the official IAPT measure for recovery.

Ethics and dissemination

The study received the following approvals: South Central – Berkshire Research Ethics Committee on 28/04/2020 (REC reference 20/SC/0135) and HRA on 23/06/2020. An amendment was approved by the Ethics Committee on 01/10/2020 and HRA on 27/10/2020. Results will be made available to patients and the public, the funders, stakeholders in the IAPT services and other researchers.

Trial registration number

ISRCTN93895792

Keywords: Anxiety, depression, cognitive behavioural therapy, common mental disorders, IAPT, primary care, psychosis, psychotic experiences.

For peer review only

Strengths and limitations of this study

- The TYPPEX study will provide the first multisite, stepped-wedge cluster randomised controlled trial to investigate the clinical and cost-effectiveness of an intervention in the UK Improving Access to Psychological Therapies (IAPT) programme.
- The study intervention seeks to address the currently underserved needs of an important group of people accessing IAPT services.
- The stepped-wedge cluster design allows the intervention to be rolled out over time to all potential participants, while reducing operational and logistical challenges.
- The primary limitation is potential loss to follow-up, resulting in missing data for the secondary health economics outcome. This would challenge the internal validity of the conclusions drawn from the study.

Introduction

Psychotic experiences (PE), such as attenuated and/or fragmentary paranoid beliefs and hallucinations, are relatively common in the general population, especially amongst young people.[1] Though systematic reviews and evidence synthesis indicate that 30–40% of those with intense and frequent attenuated psychotic symptoms will transition to a psychotic disorder,[2, 3] studies including individuals with shorter and less intense mental symptoms have shown that far fewer (~10%) make such a conversion.[4, 5] Nonetheless, PE still predict propensity to seek treatment from mental health services[6] and are a marker for severity of other, non-psychotic common mental disorders (CMD), particularly depression and anxiety.[4, 7, 8]

In England, the Improving Access to Psychological Therapies (IAPT) programme is the main provision for people with common mental disorders. Offering UK National Institute for Health and Care Excellence (NICE)-approved psychological therapies, the programme is predominantly based on cognitive behavioural therapy (CBT). National standards specify that 50% of IAPT service users should achieve recovery, a key performance indicator defined by reduction in scores on indices of depression and anxiety. For the first time since its inception in 2008, IAPT met this target in 2016-17, with 51% of people nationally being considered recovered by the end of treatment.[9]

Evidence suggests that at least one in four people treated by IAPT may have a common mental disorder that includes psychotic experiences (CMD-PE). These individuals are at increased risk of not demonstrating recovery.[5, 10] Currently, IAPT neither screens for, nor offers specific treatments for CMD-PE. Treatment protocols focus exclusively on mood disturbance, leaving PE undetected and untreated. Given the complexity and co-morbidity of CMD-PE, standard treatment interventions are likely to be sub-optimal.

Though some effective psychological treatments for PE exist, such as CBT for at-risk mental states,[11, 12] they are dispersed throughout various service settings, and as such are

For peer review only

inadequate to assess or treat the whole condition of CMD-PE. An innovative National Institute for Health Research (NIHR)-funded programme – Tailoring evidence-based psychological therapY for People with common mental disorder including Psychotic EXperiences (TYPPEX) – is targeting this clinical gap. It seeks to adapt and assemble available treatment options for CMD-PE into a practical therapeutic toolbox for cognitive behavioural therapists in IAPT known as CBT Tailoring for Severity (CBT-ts), rolled out via an enhanced training package and supervision programme.

A single-arm, three-site feasibility study (NHS REC reference 19/SC/0077) demonstrated the successful delivery of CBT-ts to 31 therapists and outcome data collection for 153 CAPE+ patients on their caseload, and provided qualitative information used to refine the training intervention (study report available by request from the authors).

This protocol paper describes the TYPPEX stepped wedge cluster Randomised Controlled Trial (TYPPEX swcRCT), which aims to determine the clinical and cost-effectiveness of CBT-ts versus IAPT standard care (SC) in IAPT service users with CMD and also PE, as determined by the Community Assessment of Psychic Experiences - Positive 15-items Scale (CAPE-P15)[13]. This pragmatic trial will be the first to identify and treat people with CMD-PE in IAPT services. It will provide valuable evidence for the development of interventions for this important and underserved population, while adding to the body of knowledge about design and conduct of randomised controlled trials in the IAPT setting.

Methods

This protocol is reported in accordance with Standard Protocol Items: Recommendations for Interventional Trials (SPIRIT) guidance[14] and refers to study protocol version 2.0 dated 4th of August 2020, which was the first version to be implemented. Amendments made to the protocol since commencement of the trial are shown in Table 1. Participant timelines and measures are summarised in Table 2.

-- Table 1 about here –

-- Table 2 about here –

Public and Patient Involvement

An experienced public and patient involvement (PPI) representative contributed significantly to the development of the PPI strategy set out in the grant application and continues to have study oversight as a member of the steering committee. The submission was endorsed by a co-applicant with relevant lived and academic experience and a PPI coordinator with lived experience was employed to facilitate PPI activity.

For peer review only

At the planning stage a Lived Experience Advisory Panel ('LEAP') was convened comprising 11 people with lived experience including diverse community members. The LEAP influenced some research questions extending the scope of a systematic review within the research programme [15] and informing qualitative interview questions.

Since TYPPEX is a pragmatic trial, the study design is largely dictated according to the way that IAPT services operate. The primary outcome measure is a national metric of 'recovery' derived from routinely collected IAPT data, and is not open to alteration. LEAP members positively influenced secondary outcome measures including introducing the CAPE-P15 at final follow up.

LEAP involvement in the development of the intervention (CBT-ts) was highly influential, for example in review of the training resources and ideas about language and delivery. All patient-facing resources are produced in partnership with the LEAP, who have been invaluable in raising issues around acceptability and accessibility. The panel reviewed the feasibility study protocol and gave feedback on adaptations to remote working for the main trial. All ethical submissions undergo thorough PPI review with strong focus on acceptability of consent processes.

PPI representation from IAPT services was not initially possible since primary care services do not routinely screen for PE. However, after the trial's feasibility study, we have expanded involvement to include three people recruited for such study, who have lived experience that matches our target population. We have also expanded involvement to include four IAPT CBT therapists, also drawn from the feasibility study, to ensure acceptability and usability of CBT-ts and research methods within an IAPT setting, together contributing an additional seven perspectives.

People with lived experience will be involved in qualitative data interpretation to provide context and sense-checking of themes and anonymised service-user quotes as part of the process evaluation. LEAP members have co-authored papers and presented posters at public events and dissemination activity will continue to be informed and enhanced by people with lived experience.

PPI will remain reflective and responsive to new insights and the needs of the study, working to the NIHR INVOLVE's Standards for Public Involvement.[16]

Study Setting

The IAPT programme in England began in 2008 with the aim of improving access to evidence-based psychological treatment for CMD, particularly depression and anxiety. It sought to increase public access to National Institute for Health and Care Excellence (NICE)-approved psychological therapies, predominantly cognitive behavioural therapy (CBT), by offering flexible referral routes, including self-referral. The programme has continued to expand over time and currently assesses over 1.3 million people with CMD annually, delivering therapy to approximately 550,000.[9] NHS England has committed to further expansion of the programme, aiming to see 1.9 million patients annually by 2024.[17].

For peer review only

The programme provides treatment for CMD for people aged 17 or over. Its pathways involve a stepped care model, providing steps 2 and 3 of the National Institute for Clinical Excellence (NICE) five-step approach to the treatment of CMD. At step 2 in the IAPT programme, large numbers of service users, with less severe conditions may be allocated to a single therapist. These service users may receive a variety of interventions, including brief face-to-face therapy, telephone support, computerised CBT or guided self-help. At step 3, highly trained CBT therapists manage smaller caseloads of people with more severe CMD. These therapists use more intensive treatment methods, delivering up to 20 individual face-to-face psychological therapy sessions. This clinical setting is immediately below psychiatric treatment by secondary mental health services (NICE step 4.

Step 3 IAPT CBT therapists will have completed a one-year postgraduate diploma (PG Dip) in CBT accredited by the British Association for Behavioural and Cognitive Psychotherapies (BABCP). Entry requirements for the PG Dip include a secured work placement for clinical practice and a BABCP recognised core profession, such as mental health nursing or counselling psychology.[18]

Trial design and Participants

The TYPPEX study is a pragmatic, multi-centre stepped wedge cluster Randomised Controlled Trial (swcRCT) with nested health economic and process evaluations. It involves eight NICE step-3 IAPT teams (clusters) of CBT therapists across three mental health trusts in England: Cambridgeshire and Peterborough NHS Foundation Trust (CPFT), Norfolk and Suffolk NHS Foundation Trust (NSFT) and Sussex Partnership NHS Foundation Trust (SPFT). Each trust (organisational unit within the National Health Services of England and Wales) serves a mixed urban and rural population with a wide socio-economic range, including some high levels of deprivation, covering a total population of approximately 2.8 million.

Clusters will be randomised to different sequences rather than, strictly speaking, different arms. The sequences dictate the order in which each cluster switches from the control to intervention condition. TYPPEX swcRCT has five measurement periods and four steps, with two clusters switching to the intervention condition at each step. As the intervention is cluster randomised at the level of IAPT team, and the delivery of therapy by these teams may be subject to regional variation, each cluster will act as its own internal control. All clusters will start in the control condition and move to the intervention condition (see Figure 1).

The primary outcome of this study will be the proportion of eligible individuals with CMD-PE, as determined by the CAPE-P15 cut-off of 1.47[19], who have recovered by the end of treatment, measured via pseudonymous clinical outcome data obtained from IAPT clinical records and IAPT-defined metrics of recovery, as specified in Table 2.

-- Figure 1 about here –

Eligibility criteria

IAPT therapist inclusion criteria are: (1) qualified IAPT CBT therapist, and (2) willing and able to provide informed consent to receive CBT-ts training and supervision. IAPT therapist exclusion criteria are: (1) does not deliver CBT therapy in current role, (2) works across more than one locality IAPT team (to avoid potential contamination e.g. via team meetings, shared office space), and (3) participated in an earlier TYPPEX feasibility study.

Service user participants are those individuals accepted onto the NICE step-3 IAPT caseload for participating therapists and meeting the study eligibility criteria, which are: (1) accepted onto the IAPT caseload for therapy and therefore meet service-specific inclusion criteria to access IAPT treatment (irrespective of whether service has been accessed previously or not), (2) meet current criteria for IAPT CBT treatment, and (3) assessed for PE, according to the CAPE-P15 questionnaire in their clinical record. Additional inclusion criteria for health economics sub-study only: (4) presence of PE according to the CAPE-P15 score, (5) in the judgement of the treating therapist has sufficient proficiency in English to complete research questionnaires, and (6) able and willing to provide written informed consent.

IAPT service user exclusion criteria are: (1) presence of mental disorder, such as complex and severe depression, based on standard IAPT assessment meriting routine referral to NICE step 4treatment, i.e. to secondary mental health services.

Participant withdrawal

The right of any participant – therapist or service user – to discontinue participation without giving any reason will be respected. People will remain free to withdraw from the trial at any point without prejudicing their further treatment or, for therapists, employment. Reason for withdrawal, if given, will be recorded. Service users who have refused or withdrawn consent for data sharing will be excluded from pseudonymous routine data collection.

Randomisation

IAPT therapists from local teams within participating sites will be recruited and grouped as study clusters before randomisation (without stratification by cluster characteristics) to the control-intervention sequences via computer written code. In most cases, clusters will be formed from a single IAPT team. Some teams may have fewer IAPT CBT therapists available for training due to empty therapist posts or service demands. In these cases, two local IAPT teams will be merged to create a cluster. Once clusters are allocated, IAPT service managers will be approached, and CBT-ts training courses arranged in line with each cluster randomisation point.

Blinding

The study will be conducted in a controlled open format with unblinded assessment; neither the research team nor the therapists that form the study clusters can be blind to the

For peer review only

intervention. IAPT service users will be made aware of the study via an information leaflet given during screening but will not know whether their therapist has received CBT-ts training.

Sample size

CBT-ts training will be delivered to step-3 CBT therapists in eight clusters, randomised at four steps. In each cluster, approximately 7-10 therapists will receive training, with the intention for at least five therapists to deliver therapy throughout each cluster-period, to allow for inevitable therapist turnover and sickness.

Based on our prevalence studies of CMD-PE in IAPT [5, 10] and considering average caseloads of 15-20 services users per therapist over six months, each therapist will deliver therapy to a minimum of three people. Pragmatically, the total number of eligible service users receiving therapy from a participating therapist during the whole trial may be higher, but only control service users who complete therapy during their cluster's control phase, and intervention service users who complete therapy prior to the end of the intervention phase, will be included in the primary outcome analysis. Based on results from our previous TYPPEX feasibility work, we expect that 6-month steps would be sufficient for this.

All service users will be identified as having PE with the CAPE-P15 score threshold of 1.47[19] for both frequency of and distress associated to psychotic experiences at baseline, resulting in a minimum of 15 service users per cluster period. Eight clusters and four randomisation steps result in 20 control condition cluster-periods and 20 intervention condition cluster-periods. 300 IAPT service users will be recruited during the control condition and 300 during the intervention condition across all sites. In addition to the number of clusters and the steps presented above, we have assumed: an intra-cluster correlation coefficient of 0.05, a significance level of 0.05, and an improvement in recovery from 0.39 (control) to 0.58 (intervention) [5, 10]. Based on these assumptions, the proposed sample size will allow us to detect such a difference with just over 80% power. Although this ignores that clustering also happens at the therapist level, it has been shown that ignoring an additional, lower level of clustering is a conservative estimate of the power. This is not sensitive to the choice of intra-cluster correlation coefficient [20].

Also during the feasibility work we found that approximately 50% of the sample providing pseudonymised routine data would consent to complete the health economic measures. Thus, we aim that 150 service users will be consented to complete health economic measures during the control condition and 150 during the intervention phase across all sites.

Procedures

All IAPT service users who begin a course of CBT from the outset of the trial will be asked to complete a CAPE-P15 screening questionnaire and will receive a leaflet during their first treatment session. The leaflet explains that the trial is taking place in the service, and if eligible, their pseudonymous clinical data will be used for the research. Awareness of the

For peer review only

trial is also raised in participating sites using posters. Both the poster and leaflet provide service users with information on the local policy for how they can opt out should they wish their data not to be used for research purposes. The CAPE-P15 screening questionnaire is currently accessible for clinical information as part of routine service use for participating sites.

IAPT service users with PE according to the CAPE-P15 will be asked by their therapist to provide consent to be contacted about participating in the health economic data collection, since this data cannot be collected from clinical records. If consent is obtained, the service user will be sent a participant information sheet and an electronic consent form by email (or paper versions by post) prior to the following treatment session, followed by baseline health economics measures.

Recruited service users will receive standard IAPT care from a participating therapist (control condition) or standard IAPT care from a participating therapist following CBT-ts training (intervention condition). They will be contacted by email or post for follow-up health economics data collection at 3, 6, 9, and 12 months after baseline.

Intervention

CBT-Tailoring for severity (CBT-ts)

CBT-ts is a team-level service improvement intervention. It aims to enhance existing CBT skills of NICE step-3 IAPT CBT therapists and is not a novel therapeutic intervention. Therefore, it is important to highlight that all IAPT service-users receiving CBT under the care of a CBT-ts trained therapist, including those who do not have CMD-PE, may be exposed to the effects of the training.

CBT-ts is based on the CBT framework currently used nationally in IAPT but includes adaptations to existing interventions to specifically address PE. The training takes a knowledge, skills and attitude focus enhancing existing knowledge of CBT. It includes three modules delivered online as workshops supplemented by a blended learning package with access to an online network and provision of written resources. Therapists are provided with a single pack of editable templates, which they may or may not use in their clinical practice.

The structured training programme includes:

Module 1:

- Introduction to the concept of CMD-PE and the relationship between CMD and PE.
- Prevalence and impact in IAPT services.
- Identification of PE.
- Normalisation and validation of CMD-PE.

Module 2:

For peer review only

- Incorporating assessing PE into existing assessments within CBT.
- Suitability for IAPT.
- Understanding CMD-PE maintenance cycles.
- Approaches to the therapeutic conceptualisation and formulation of CMD-PE including co-morbidity.

Module 3:

- Adaptations to existing cognitive behavioural strategies and the application of these to promote the therapeutic change, using change methodologies, such as metaphors, exposure, cognitive restructuring and behavioural experiments.
- Relapse prevention.

Training is supported by six CBT-ts supervision sessions, which commence one month post training and are subsequently delivered monthly, either online or face-to-face. Following completion of training, CBT therapists will identify people with CMD-PE according to the CAPE-P15, and treat them in line with current CBT interventions, incorporating their enhanced skills, as necessary. Treatment duration is non-prescriptive and expected to range between 8 to 14 sessions dependent upon therapists' clinical decisions. Treatment duration is not expected to exceed 20 weekly sessions, in line with current standard guidance in IAPT services, nationally.

Standard care

The control comparator is standard CBT care delivered within IAPT services prior to CBT-ts training. CBT is delivered as part of range of interventions offered at step 3 within the NICE stepped care model. CBT treatment protocols are delivered in accordance with NICE guidance for people with CMD. Treatment duration varies according to presenting problem and minimum dose necessary to achieve recovery, but NICE recommends individuals should receive up to 20 weekly sessions unless they recover beforehand (https://www.nice.org.uk/guidance/cg123/chapter/1-guidance).

Outcomes

A summary of study outcomes is shown in Table 3, and schedules of outcome measures for service users and therapists are provided in Tables 2 and 4.

-- Table 3 about here –

-- Table 4 about here –

Process evaluation

The aim of the process evaluation is to assess the views of all stakeholders involved in the experience and delivery of the TYPPEX swcRCT and CBT-ts, and to investigate the implementation of CBT-ts as designed, including any influences on uptake, delivery, and fidelity. An overview of the key themes explored with each stakeholder group is given in Table 5.

-- Table 5 about here –

The aim of sampling for the process evaluation will be to achieve theoretical saturation [21], where further new data is unlikely to achieve further insights or to add to analytic depth. Up to 32 service users across the eight clusters with scores of 1.47 and above on the CAPE-P15 and who have completed at least two CBT treatment sessions with a CBT-ts trained therapist will be recruited including those who have completed treatment, those who are still undergoing therapy and those who dropped out of treatment. Where possible, purposive sampling will be used to ensure diversity of gender and ethnicity and exposure to number of sessions of therapy.

Between 16 and 24 IAPT therapists (with a minimum of two per cluster) who completed CBT-ts training and subsequently delivered at least three treatment sessions to a minimum of two service-users scoring 1.47 and above on the CAPE-P15 will be recruited. Additionally, we will conduct 18-25 interviews with IAPT clinical supervisors/managers from participating IAPT teams who line manage or supervise a CBT-ts trained therapist, senior non-clinical NHS managers with responsibility for delivery of IAPT services at Trust level or responsibility for commissioning IAPT services, and members of the trial team and collaborators who contributed to the design, development, or implementation of TYPPEX. In all cases, purposive sampling will be used to ensure representation of a wide range of perspectives and experiences.

Data management

Pseudonymised outcome data will be received by Norwich Clinical Trials Unit from sites and uploaded to a central study database stored on servers based at the University of East Anglia. Health economic questionnaire data will be collected using REDCap data collection software (https://www.project-redcap.org). Questionnaires will be completed online or by post by service users. Pseudonymised clinical data will be linked to consented participants' health economics data held by NCTU.

Data collection will include retrospectively recorded IAPT data from 23rd March 2020 until trial start. This data, covering the period of UK lockdown during the COVID-19 pandemic, will provide information on the nature of IAPT service provision during this time, and any

For peer review only - http://bmjopen.bmj.com/site/about/guidelines.xhtml

changes in baseline morbidity. The aim of the data collection is to provide insight into standard of care and baseline severity during the pandemic, to assist decision-making by the trial oversight committees.

Data will be handled in accordance with a data management plan and comply with the principles of the International Conference on Harmonisation Good Clinical Practice, within the Standard Operating Procedures for Data Management in NCTU and where appropriate with UEA IT procedures. Interview transcriptions will be anonymised in adherence with the "Guidance on Anonymisation" issued by the UK Data Service (https://www.ukdataservice.ac.uk/manage-data/legal-ethical/anonymisation.aspx). Anonymised data will be stored in password-protected files on the firewall-protected University of Cambridge servers.

Statistical analyses

The primary analysis will compare IAPT-defined recovery of service users with CMD-PE as determined by the CAPE-P15 cut-off of 1.47 in the control phase with those in the intervention phase (post-training). Recovery proportions and means will be compared at individual therapist and team level after adjusting for the stepped-wedge cluster design. Adjustment will also be made for the baseline characteristics and other covariates. Time will be included as a set of indicator variables.

Analyses of clinical effectiveness will be based on the Intention to Treat (ITT) population with all available follow-up data from all new service users with CMD-PE on the caseload of participating therapists, controlling for baseline scores on depression and anxiety disorder specific measures. The primary ITT analysis is intended to provide inferences regarding the effectiveness of the intervention overall. It does not provide inferences regarding the causal effect of the intervention itself, but on the intervention as deployed in 'real life', therefore compliance information is not necessary to ensure that the ITT analysis is valid. As soon as a therapist has their first session of CBT-ts training, they begin to practise differently, so this date will mark the beginning of their intervention phase.

A logistic mixed effects regression model will be used for the primary outcome analysis. The cluster effect by both the therapist team and therapist will be considered in the multilevel analysis. Adjustment for potential prognostic variables will be agreed prior to analysis between the trial statisticians and the Chief Investigator.

Intra-cluster correlation coefficients and 95% confidence intervals will be reported for the primary and secondary outcomes. All analyses will be two-tailed and at the 5% level of significance.

Summary statistics of baseline characteristics at the cluster and the individual level will be reported where appropriate. Analyses will be conducted in Stata or R. Publication of results will include a participants/cluster flow diagram, and results will be reported according to the CONSORT statement for stepped wedge designs.

For peer review only

Economic analysis

The economic evaluation will be conducted covering the period from baseline to 12-month follow-up and will take the NHS/Personal Social Services perspective preferred by NICE[22]. The intervention will be costed taking a bottom-up (micro-costing) approach[23] using data collected on face-to-face contacts from IAPT records and accounting for the ratio of direct face-to-face to indirect non-face-to-face time. Data on indirect time, including preparation and supervision, will be collected directly from the therapists. Details of resources required to deliver the CBT-ts training and supervision will be provided by the TYPPEX research team. Use of all other health and social care services will be collected via a modified version of the Adult Service Use Schedule for Early Intervention (EI-ADSUS; Table 1) collected using REDCap data collection software at baseline (covering the last 3 months) and 3-, 6-, 9-, and 12-months (covering the period since the last interview). Resource use data will be combined with nationally applicable unit costs.[24, 25] Productivity losses (applicable to sensitivity analyses only) will be calculated using the human capital approach by multiplying days off work for mental health reasons by the national average wage rate.[26]

Our primary economic analysis will be a cost-utility analysis using Quality Adjusted Life Years (QALYs) derived from the EuroQol measure of health-related quality of life (EQ-5D) based on complete case data. The EQ-5D-5L has been proposed as being more sensitive to change in mental health populations compared to the EQ-5D-3L.[27] However, there is little evidence to support this hypothesis. Therefore, we will compare the psychometric properties of the 3L and 5L versions. The version of the EQ-5D used in this analysis will be dependent on analyses comparing the two. QALYs will be calculated by applying appropriate utility weights to EQ-5D health states[28] and using the total area under the curve approach with linear interpolation between assessment points.[29] A secondary analysis will additionally explore cost-effectiveness using the primary outcome measure: IAPT-defined recovery.

Costs and outcomes will be compared at the final follow-up point and presented as mean values by trial phase with standard deviations. Mean differences in costs and 95% confidence intervals will be obtained by nonparametric bootstrap regressions to account for the non-normal distribution commonly found in economic data[30]. Incremental cost-effectiveness ratios (ICERs) will be calculated. Uncertainty will be explored using cost-effectiveness planes and cost-effectiveness acceptability curves (CEACs) based on the net-benefit approach.[31] These curves are an alternative to confidence intervals around incremental cost-effectiveness ratios and show the probability that one intervention is cost effective compared to the other for a range of values that a decision maker would be willing to pay for an additional unit of an outcome. To provide more relevant treatment-effect estimates, all economic analyses will include adjustment for stepped-wedge cluster design, relevant baseline characteristics and other covariates[32], in line with the clinical analyses.

Sensitivity analyses will be conducted to explore the impact of missing data (using multiple imputation) and to explore cost-effectiveness from a broader perspective, including productivity losses because of time off work due to illness. Additionally, to assess the impact of missing data, we will compare those with economic data to the full sample included in

For peer review only

the main clinical analysis to examine any potential biases in terms of demographic and clinical factors.

Process evaluation analysis

Analysis of process evaluation data will be based on the constant comparative method.[33,34] Practically, this will be achieved through multiple levels of analysis, systematically comparing data on different questions and from different sources (quantitative and qualitative) to achieve thematically ordered synthesis. Data analysis will take place after each stage of data collection so the analysis from each stage can inform data collection in the next. Some data analysis will also take place in parallel. The data will initially be subject to open coding using a combination of pre-selected questions and sensitising constructs identified from the literature. Codes will be increasingly grouped into higher-order explanatory categories through comparison and refinement in rounds of discussion and sense-making. A set of overarching thematic categories will be agreed and used as a framework for further, more deductive, coding across the whole data set.

Trial Status

The trial was opened to IAPT service user recruitment in March 2021, with the first randomisation point scheduled for September 2021. Recruitment will continue until September 2023. The final 12 month follow-up is expected to be completed by October 2024.

Ethics and dissemination

This study was approved by the South Central – Berkshire Research Ethics Committee (REC reference 20/SC/0135 on 28/04/2020 and HRA on 23/06/2020). Subsequent amendments are shown in Table 1.

The study is jointly sponsored by the University of Cambridge and Cambridgeshire and Peterborough NHS Foundation Trust. It was adopted onto the NIHR trial portfolio on 16/04/2020.

CBT-ts, including therapist training and supervision, is low risk for all participants. IAPT service users scoring above the CAPE-P15 thresholds for PE on whom data is being pseudonymously collected are under the care of the IAPT service for the duration of their participation and are subject to normal IAPT safety procedures. No specific risks, untoward incidents or adverse events related to CBT-ts are anticipated as the intervention aims to up-skill therapists rather than offering a new therapeutic intervention. This approach was supported by the results of a feasibility study as part of the wider TYPPEX research programme, during which no related serious adverse events were reported.

Safety outcome variables will be collected retrospectively each month from the routinely collected pseudonymous data. Aggregated safety information will be reported to the TYPPEX Data Monitoring and Ethics Committee (DMEC) throughout the randomisation condition. In

For peer review only

addition, Principal Investigators, IAPT managers and therapists will be requested to report complaints relating to TYPPEX by eligible participants to the trial team.

The clinical data generated during therapy (CAPE-P15, GAD-7, PHQ-9 and ADSM) is recorded as part of routine care and is used by this trial via an opt-out consent process. This is designed to remove the burden on IAPT therapists with respect to recruitment, and to ensure a representative sample.

A sub-group of service users are consented via an opt-in process to provide additional data outside of the clinic setting. This includes cost and quality of life health economic data, and follow-up clinical data at 12 months (see Table 2). This data will be linked with their IAPT clinical record.

Results from this trial will be presented at national and international conferences, including the Schizophrenia International Research Society (SIRS) Conference and IEPA *Early Intervention in Mental Health* Conference. Dissemination will also occur through the submission of a primary article on the outcomes and several subsequent articles considering the effects of the intervention stratified by service user and therapist variables. Primary outcomes will be disseminated more widely as part of a theory-driven implementation plan, developing a community of practise (CoP) including patients, the public, economists and systems engineers experienced in care pathway design and evaluation, in addition to stakeholders in the IAPT services.

The training materials will be shared electronically and will be widely available to the NHS.

Contributors

PBJ and JP are co-CIs for and designed this trial. P-AA is the trial manager. CK was manager for the wider research programme. UP led the design and development of the intervention, supported by DAR. JH, NR, NM, JC, MP, NG, HM, DF and PF advised on the development of the intervention. TK leads on the PPI strategy. FS supports all elements of the PPI strategy and, with DAR, also delivers research assistant functions for the trial. AC and MK contributed to the trial design and oversee the statistical analysis. JS advises on statistical matters. MH and SB lead the health economic component of the trial. MD-W oversees qualitative analyses and process evaluation. ML and MP are responsible for trial data management. AMS oversees all operations at the Clinical Trials Unit. P-AA, CK, MH, AC, MK and JP wrote the first draft of this manuscript. All authors reviewed, contributed to, and commented on the final draft.

Funding

This paper presents independent research funded by the National Institute for Health Research (NIHR) under its Programme Grants for Applied Research Programme (Reference Number RP-PG-0616-20003). The views expressed are those of the author(s) and not necessarily those of the NHS, the NIHR or the Department of Health.

For peer review only

The funding body had no role in study design; collection, analysis, or interpretation of data; the writing of the report; or decision to submit the paper. The first author had full access to all data and had final responsibility for the decision to submit for publication.

Competing interests

None declared.

References

1. Linscott RJ, van Os J. An updated and conservative systematic review and meta-analysis of epidemiological evidence on psychotic experiences in children and adults: on the pathway from proneness to persistence to dimensional expression across mental disorders. *Psychol Med* 2013;43(6):1133-49 doi:10.1017/S0033291712001626.

2. Fusar-Poli P, Bonoldi I, Yung AR, Borgwardt S, et al. Predicting psychosis: meta-analysis of transition outcomes in individuals at high clinical risk. *Arch Gen Psychiatry* 2012;69(3):220-9 doi:10.1001/archgenpsychiatry.2011.1472.

3. Addington J, Epstein I, Liu L, et al. A randomized controlled trial of cognitive behavioral therapy for individuals at clinical high risk of psychosis. *Schizophr Res* 2011;125(1):54-61 doi:10.1016/j.schres.2010.10.015.

4. Hui, C, Morcillo, C, Russo, DA, et al. Psychiatric morbidity, functioning and quality of life in young people at clinical high risk for psychosis. *Schizophr Res* 2013;148(1–3):175–180.

5. Perez J, Russo DA, Stochl J, et al. Common mental disorder including psychotic experiences: trailblazing a new recovery pathway within the Improving Access to Psychological Therapies programme in England. *Early Interv Psychia* 2017;12(3):497–504 doi:https://doi.org/10.1111/eip.12434.

6. Devylder JE, Oh HY, Corcoran CM, et al. Treatment seeking an unmet need for care among persons reporting psychosis-like experiences. *Psychiatr Serv* 2014;65(6):774–780 doi:https://doi.org/10.1176/appi.ps.201300254.

7. Wigman JT, Nierop M.V, Vollebergh WA, et al. Evidence that psychotic symptoms are prevalent in disorders of anxiety and depression, impacting on illness onset, risk, and severity–implications for diagnosis and ultra-high risk research. *Schizophr Bull* 2012;38(2):247–257 doi:https://doi.org/10.1093/schbul/sbr196.

8. Stochl J, Khandaker GM, Lewis G, et al. Mood, anxiety and psychotic phenomena measure a common psychopathological factor. *Psychol Med* 2015;45(7):1–11. doi:10.1017/S003329171400261X.

9. NHS Digital. Psychological Therapies, Annual report on the use of IAPT services - England, 2017-18 (online). https://digital.nhs.uk/data-and-information/publications/statistical/psychological-therapies-annual-reports-on-the-use-of-iapt-services/annual-report-2017---18 (accessed September 2020).

10. Knight C, Russo D, Stochl J, et al. Prevalence of and recovery from common mental disorder including psychotic experiences in the UK Primary Care Improving Access to Psychological Therapies (IAPT) Programme. *J Affect Disord* 2020;(1):272:84-90 doi:10.1016/j.jad.2020.04.015.

For peer review only

11. Morrison AP, French P, Stewart SL, Birchwood M, Fowler D, Gumley AI, Jones PB, Bentall RP, Lewis SW, Murray GK, Patterson P, Brunet K, Conroy J, Parker S, Reilly T, Byrne R, Davies LM, Dunn G. Early detection and intervention evaluation for people at risk of psychosis: multisite randomised controlled trial. *BMJ* 2012;(5)344:e2233.

12. van der Gaag M, Nieman DH, Rietdijk J, Dragt S, Ising HK, Klaassen RM, Koeter M, Cuijpers P, Wunderink L, Linszen DH. Cognitive behavioral therapy for subjects at ultrahigh risk for developing psychosis: a randomized controlled clinical trial. *Schizophr Bull* 2012;38(6):1180-8.

13. Capra C, Kavanagh DJ, Hides L, et al. Current CAPE-15: a measure of recent psychotic-like experiences and associated distress. *Early Interv Psychia* 2017;11(5):411–417 doi: https://doi.org/10.1111/eip.12245.

14. Chan A-W, Tetzlaff JM, Altman DG, et al. SPIRIT 2013 Statement: Defining standard protocol items for clinical trials. *Ann Intern Med* 2013;158(3):200-207.

15. Soneson E, Russo D, Stochl J, et al. Psychological interventions for people with psychotic experiences: A systematic review and meta-analysis of controlled and uncontrolled effectiveness and economic studies. *Aust N Z J Psychiatry* 2020;54(7):673-695. doi: 10.1177/0004867420913118.

16. NIHR Involve. National Standards for Public Involvement in Research V1 March 2018 (online). https://www.invo.org.uk/posttypepublication/national-standards-for-public-involvement/ (accessed November 2020).

17. Clark DM. IAPT at 10: achievements and challenges, 2019 (online). https://www.england.nhs.uk/blog/iapt-at-10-achievements-and-challenges/ (accessed September 2019).

18. British Association for Behavioural & Cognitive Psychotherapies (BABCP) training in cognitive behavioural therapy: https://babcp.com/Careers/Training-in-CBT (accessed 22/04/2022)

19. Bukenaite A, Stochl J, Mossaheb N, et al. Usefulness of the CAPE-P15 for detecting people at ultra-high risk for psychosis: Psychometric properties and cut-off values. *Schizophr Res* 2017;189:69-74. doi: 10.1016/j.schres.2017.02.017.

20. Hussey MA, Hughes JP. Design and analysis of stepped wedge cluster randomized trials. *Contemp Clin Trials* 2007;28(2):182-91.

21. Guest G, Namey E, Chen M. A simple method to assess and report thematic saturation in qualitative research. PLoS One. 2020;15(5):e023207622. National Institute for Health and Care Excellence. Guide to the methods of technology appraisal 2013. London: NICE 2013.

23. Drummond MF, Sculpher MJ, Torrance GW, et al. Methods for the economic evaluation of health care programmes. New York: Oxford University Press 2005.

24. Curtis L, Burns A. Unit Costs of Health and Social Care 2018. University of Kent, Canterbury: Personal Social Services Research Unit 2018.

25. NHS improvement. 2017/18 Reference Costs (online). https://webarchive.nationalarchives.gov.uk/20200501111106/https://improvement.nhs.uk/documents/6468/201718_reference_costs_data_and_guidance.zip/ (accessed March 2020).

26. Koopmanschap MA, Rutten FF. A practical guide for calculating indirect costs of disease. *Pharmacoeconomics* 1996 Nov;10(5):460-6. doi:10.2165/00019053-199610050-00003.

For peer review only

27. Camacho EM, Shields G, Lovell K, et al. A (five-) level playing field for mental health conditions?: exploratory analysis of EQ-5D-5L-derived utility values. *Qual Life Res* 2018;27(3):717-24.

28. Dolan P, Gudex C, Kind P, et al. A social tariff for EuroQol: results from a UK general population survey. York: University of York, Centre for Health Economics 1995.

29. Manca A, Hawkins N, Schulpher MJ. Estimating mean QALYs in trial-based cost-effectiveness analysis: the importance of controlling for baseline utility. *Health Econ* 2005;14(5): 487-496.

30. Thompson SG, Barber JA. How should cost data in pragmatic randomised trials be analysed? *BMJ*. 2000;320:1197-200.

31. Briggs AH. A Bayesian approach to stochastic cost-effectiveness analysis. *Health Econ* 1999;8:257-261.

32. Assmann SF, Pocock SJ, Enos LE, et al. Subgroup analysis and other (mis) uses of baseline data in clinical trials. *Lancet*. 2000 Mar 25;355(9209):1064-9.

33. Charmaz K. Constructing grounded theory. London: Sage Publications 2014.

34. Charmaz K. Grounded theory: Objectivist and constructivist methods. In: Denzin NK, Lincoln YS, eds. Strategies for qualitative inquiry (2nd ed.). Thousand Oaks, CA: Sage; 2003:249-291.

35. Kroenke K, Spitzer RL, Williams JB. The PHQ-9: validity of a brief depression severity measure. *J Gen Intern Med* 2001;16(9):606–613. doi:10.1046/j.1525-1497.2001.016009606.x.

36. Spitzer RL, Kroenke K, Williams JB, et al. A brief measure for assessing generalized anxiety disorder: the GAD-7. *Arch Intern Med* 2006;166(10):1092–1097 doi:https://doi.org/10.1001/archinte.166.10.1092.

37. Perez J, Jin H, Russo DA, et al. Clinical and cost-effectiveness of tailored, intensive liaison between primary and secondary care to detect individuals at risk of a first psychotic illness: a theory-based, cluster-randomised controlled trial. *Lancet Psychiatry*. 2015;2(11):984-93.

38. The National Collaborating Centre for Mental Health, 2018. The improving access to psychological therapies manual (online). https://www.rcpsych.ac.uk/docs/default-source/improving-care/nccmh/the-iapt-manual–final–republished-7-3-18.pdf?sfvrsn= a192d1af_0 (accessed September 2020).

39. Knight C, Russo D, Stochl J, et al. More sensitive identification of psychotic experiences in common mental disorder by primary mental healthcare services - effect on prevalence and recovery: casting the net wider. *BJPsych Open* 2020;6(6):e136.

40. Group TE. EuroQol-a new facility for the measurement of health-related quality of life. *Health Policy* 1990;16(3):199-208.

41. Herdman M, Gudex C, Lloyd A, et al. Development and preliminary testing of the new five-level version of EQ-5D (EQ-5D-5L). *Qual Life Res* 2011;20(10):1727-36.

42. Stochl J, Croudace T, Perez J, et al. Usefulness of EQ-5D for evaluation of health-related quality of life in young adults with first-episode psychosis. *Qual Life Res* 2013;22(5):1055-63.

43. Lovett R, Cooper S, NICE Science policy and research programme. NICE to support new valuation study for England for EQ-5D-5L questionnaire (online). https://www.nice.org.uk/news/blog/nice-to-support-new-valuation-study-for-england-for-eq-5d-5l-questionnaire (accessed May 2021)

For peer review only

Figure legend: Figure 1. Stepped wedge trial diagram

Table 1: Summary of ethical amendments

Protocol	Primary reasons for amendment
2.0, 04/08/2020	• Addition of secondary clinical outcomes (Reliable Recovery and Reliable Improvement).<br>• Addition of retrospective pseudonymous clinical data collection from the beginning of UK lockdown due to COVID-19, until randomisation.<br>• Intervention adaptation: training to be delivered either online or face-to-face.<br>• Changes to the service user consent process: moving from full consent on tablet devices to consent to contact followed by full consent online at home.
3.0, 12/05/2021	• Video conferencing e.g. Zoom included as an option for Process Evaluation interviews.<br>• Removal of restriction of only teams from the same NHS Trust merging to form a cluster.

For peer review only

Table 3: Summary of study outcomes

Category	Measure	Primary/Secondary	Details
Service User Recovery	IAPT-defined 'Recovery'	Primary	The primary outcome is the proportion of IAPT service-users with CAPE-P15 score of 1.47 or above who have recovered by the end of treatment. Recovery is a national IAPT programme performance metric, with an individual deemed recovered if they scored above the clinical cut-off on the PHQ-9[35] and/or GAD-7[36] before treatment, i.e. 10 and 8 respectively, and below the clinical cut-off at the end of treatment on both measures.[37] If recorded, an anxiety disorder specific measure (ADSM) is used in place of the GAD-7 if that is the focus of treatment.[38]
	IAPT-defined 'Recovery'	Secondary	As for the primary outcome, but for IAPT service-users reaching the lower threshold score for CMD-PE (CAPE-P15 score of 1.30 and above).[39]
	IAPT-defined 'Reliable Improvement'	Secondary	Service users are considered reliably improved if they show any improvement in scores on the appropriate outcome measures between pre and post treatment, that exceeds the measurement error of the scales.[38]
	IAPT-defined 'Reliable Recovery'	Secondary	Service users are considered reliably recovered if they meet both criteria for Reliable Improvement and for Recovery.[38]
Health economic measures	Modified Adult Service Use Schedule for Early Intervention (EI-ADSUS)	Secondary	Individual-level resource use will be measured using a modified version of Adult Service Use Schedule for Early Intervention (EI-ADSUS). This was developed in previous research with similar populations[37] and adapted for electronic participant self-completion. It will include all-cause hospital and community-based health and social care resource use as well as information on time off work due to mental health.
	EuroQol measure of health-related quality of life 3 level (EQ-5D-3L)	Secondary	Health related quality of life will be measured using the EQ-5D-3L, a generic, preference-based measure based on five dimensions (mobility, self-care, usual activities, pain/discomfort, and anxiety/depression). Each dimension is rated on three levels (no problems, some problems, and severe problems).[40]
	EuroQol measure of health-related	Secondary	The EQ-5D-5L is also a health-related quality of life measure based on five dimensions (mobility, self-care, usual activities, pain/discomfort and

For peer review only

	quality of life 5 level (EQ-5D-5L)		anxiety/depression), but each dimension is rated on five levels (no problems, slight problems, moderate problems, severe problems, and extreme problems.[41]<br><br>It has been demonstrated that the EQ-5D-3L is a useful, valid instrument in young people with emerging psychotic symptoms.[42] However, the new five-level version, may prove more sensitive to change and is now recommended for economic evaluation by NICE[43] but has not been validated in this population. Therefore, we will test the sensitivity of the EQ-5D-5L in comparison to the EQ-5D-3L.
Therapist Adherence	Supervision checklist	Secondary	Therapist adherence measured using a supervision checklist and adherence score completed during the 6 monthly supervision sessions held after CBT training. Trial supervisors will review and collate therapy session notes and rate adherence to the CBT-ts approach.

For peer review only

Table 2: Schedule of IAPT user enrolment, interventions, and assessments.

	**STUDY PERIOD (CONTROL AND RANDOMISED CONDITIONS)**						
	Screening	Baseline	Treatment	Follow Up			
**TIMEPOINT**	**-t1***	**-t2**	**Up to 20 sessions**	**3**	**6**	**9**	**12**
**ENROLMENT**							
Eligibility screen	x						
Consent to contact (for health economics measures only)							
Informed consent (for health economics measures only)		x					
IAPT CBT standard care			—				
**ASSESSMENTS**							
PHQ-9**	x	x	X				x
GAD-7/ADSM**	x	x	X				x
EQ-5D-3L		x		x	x	x	x
EQ-5D-5L		x		x	x	x	x
EI-ADSUS		x		x	x	x	x
CAPE P-15	x						x

*The duration between the screening visit and baseline is anticipated to be 1-3 weeks, depending on typical frequency of therapy sessions in IAPT service. During this time the CAPE-P15 is scored and eligibility is confirmed. CAPE+ IAPT users are approached to participate in the health economics data collection during the baseline visit. Clinical data is obtained for all CAPE+ IAPT users unless they opt out.

**PHQ-9 and GAD-7 are routine IAPT clinical measures and are collected during every clinical contact. At 12 month follow-up, these measures will be collected via an opt-in process to provide additional data.

For peer review only

Table 4: Schedule of IAPT therapist enrolment, interventions, and assessments.

	Trial set-up	Randomisation	Control condition	Intervention condition
**IDENTIFICATION**				
IAPT teams and potential therapists identified and grouped to form clusters	x			
**ENROLMENT AND RANDOMISATION**				
Informed consent	x			
Clusters randomised		x		
**INTERVENTION**				
CBT-ts training				x
CBT-ts supervision				x
**ASSESSMENTS**				
Adherence and engagement record				x

Table 5: Key themes explored in the process evaluation for each stakeholder group

Stakeholder Group	Key Themes
Service users	• Experiences of treatment and therapist<br>• Views on improving IAPT services<br>• Views on CBT-ts
Therapist:	• Design and goals of CBT-ts<br>• Experience of the CBT-ts training<br>• Experience of delivery and fidelity
Line Managers	• Design and goals of CBT-ts<br>• Experience of the CBT-ts training<br>• Experience of delivery and fidelity at the therapist-service user level<br>• Experience of delivery and fidelity at the IAPT service level
Study Team and collaborators	• Rationale for CBT-ts<br>• Implementation and components<br>• Outcomes and measurement<br>• Modifications and changes to CBT-ts
Senior NHS Managers/Commissioners	• Relevance of CBT-ts in IAPT<br>• Implementation of CBT-ts model<br>• Negotiating change<br>• Measuring success

For peer review only - http://bmjopen.bmj.com/site/about/guidelines.xhtml

Cluster		Control	Step 1	Step 2	Step 3	Step 4
1	RANDOMISATION					I
2						I
3					I	I
4					I	I
5				I	I	I
6				I	I	I
7			I	I	I	I
8			I	I	I	I
		Months 1-6	Months 7-12	Months 13-18	Months 19-24	Months 25-30

Key:

Control phase
Intervention phase (I)

peer review only

STANDARD PROTOCOL ITEMS: RECOMMENDATIONS FOR INTERVENTIONAL TRIALS

SPIRIT 2013 Checklist: Recommended items to address in a clinical trial protocol and related documents*

Section/item	Item No	Description	Addressed on page number
**Administrative information**			
Title	1	Descriptive title identifying the study design, population, interventions, and, if applicable, trial acronym	________1______
Trial registration	2a	Trial identifier and registry name. If not yet registered, name of intended registry	________2______
	2b	All items from the World Health Organization Trial Registration Data Set	________2______
Protocol version	3	Date and version identifier	________4______
Funding	4	Sources and types of financial, material, and other support	________15______
Roles and responsibilities	5a	Names, affiliations, and roles of protocol contributors	______15______
	5b	Name and contact information for the trial sponsor	________14______
	5c	Role of study sponsor and funders, if any, in study design; collection, management, analysis, and interpretation of data; writing of the report; and the decision to submit the report for publication, including whether they will have ultimate authority over any of these activities	_____n/a_______
	5d	Composition, roles, and responsibilities of the coordinating centre, steering committee, endpoint adjudication committee, data management team, and other individuals or groups overseeing the trial, if applicable (see Item 21a for data monitoring committee)	_____n/a_______

For peer review only

**Introduction**			
Background and rationale	6a	Description of research question and justification for undertaking the trial, including summary of relevant studies (published and unpublished) examining benefits and harms for each intervention	3
	6b	Explanation for choice of comparators	6
Objectives	7	Specific objectives or hypotheses	4
Trial design	8	Description of trial design including type of trial (eg, parallel group, crossover, factorial, single group), allocation ratio, and framework (eg, superiority, equivalence, noninferiority, exploratory)	6
**Methods: Participants, interventions, and outcomes**			
Study setting	9	Description of study settings (eg, community clinic, academic hospital) and list of countries where data will be collected. Reference to where list of study sites can be obtained	5
Eligibility criteria	10	Inclusion and exclusion criteria for participants. If applicable, eligibility criteria for study centres and individuals who will perform the interventions (eg, surgeons, psychotherapists)	6
Interventions	11a	Interventions for each group with sufficient detail to allow replication, including how and when they will be administered	9
	11b	Criteria for discontinuing or modifying allocated interventions for a given trial participant (eg, drug dose change in response to harms, participant request, or improving/worsening disease)	n/a
	11c	Strategies to improve adherence to intervention protocols, and any procedures for monitoring adherence (eg, drug tablet return, laboratory tests)	10
	11d	Relevant concomitant care and interventions that are permitted or prohibited during the trial	n/a
Outcomes	12	Primary, secondary, and other outcomes, including the specific measurement variable (eg, systolic blood pressure), analysis metric (eg, change from baseline, final value, time to event), method of aggregation (eg, median, proportion), and time point for each outcome. Explanation of the clinical relevance of chosen efficacy and harm outcomes is strongly recommended	10
Participant timeline	13	Time schedule of enrolment, interventions (including any run-ins and washouts), assessments, and visits for participants. A schematic diagram is highly recommended (see Figure)	10

For peer review only - http://bmjopen.bmj.com/site/about/guidelines.xhtml

Sample size	14	Estimated number of participants needed to achieve study objectives and how it was determined, including clinical and statistical assumptions supporting any sample size calculations	_____7________
Recruitment	15	Strategies for achieving adequate participant enrolment to reach target sample size	_____8________

Methods: Assignment of interventions (for controlled trials)

Allocation:			
Sequence generation	16a	Method of generating the allocation sequence (eg, computer-generated random numbers), and list of any factors for stratification. To reduce predictability of a random sequence, details of any planned restriction (eg, blocking) should be provided in a separate document that is unavailable to those who enrol participants or assign interventions	___7__________
Allocation concealment mechanism	16b	Mechanism of implementing the allocation sequence (eg, central telephone; sequentially numbered, opaque, sealed envelopes), describing any steps to conceal the sequence until interventions are assigned	___7_________
Implementation	16c	Who will generate the allocation sequence, who will enrol participants, and who will assign participants to interventions	____8_________
Blinding (masking)	17a	Who will be blinded after assignment to interventions (eg, trial participants, care providers, outcome assessors, data analysts), and how	___n/a________
	17b	If blinded, circumstances under which unblinding is permissible, and procedure for revealing a participant's allocated intervention during the trial	____n/a_______

Methods: Data collection, management, and analysis

Data collection methods	18a	Plans for assessment and collection of outcome, baseline, and other trial data, including any related processes to promote data quality (eg, duplicate measurements, training of assessors) and a description of study instruments (eg, questionnaires, laboratory tests) along with their reliability and validity, if known. Reference to where data collection forms can be found, if not in the protocol	___4,11,12_____
	18b	Plans to promote participant retention and complete follow-up, including list of any outcome data to be collected for participants who discontinue or deviate from intervention protocols	____7________

For peer review only - http://bmjopen.bmj.com/site/about/guidelines.xhtml

Data management	19	Plans for data entry, coding, security, and storage, including any related processes to promote data quality (eg, double data entry; range checks for data values). Reference to where details of data management procedures can be found, if not in the protocol	_____11_________
Statistical methods	20a	Statistical methods for analysing primary and secondary outcomes. Reference to where other details of the statistical analysis plan can be found, if not in the protocol	______12________
	20b	Methods for any additional analyses (eg, subgroup and adjusted analyses)	______12________
	20c	Definition of analysis population relating to protocol non-adherence (eg, as randomised analysis), and any statistical methods to handle missing data (eg, multiple imputation)	______12________
**Methods: Monitoring**			
Data monitoring	21a	Composition of data monitoring committee (DMC); summary of its role and reporting structure; statement of whether it is independent from the sponsor and competing interests; and reference to where further details about its charter can be found, if not in the protocol. Alternatively, an explanation of why a DMC is not needed	______14________
	21b	Description of any interim analyses and stopping guidelines, including who will have access to these interim results and make the final decision to terminate the trial	_____-_________
Harms	22	Plans for collecting, assessing, reporting, and managing solicited and spontaneously reported adverse events and other unintended effects of trial interventions or trial conduct	______14________
Auditing	23	Frequency and procedures for auditing trial conduct, if any, and whether the process will be independent from investigators and the sponsor	_______-_______
**Ethics and dissemination**			
Research ethics approval	24	Plans for seeking research ethics committee/institutional review board (REC/IRB) approval	_____2_________
Protocol amendments	25	Plans for communicating important protocol modifications (eg, changes to eligibility criteria, outcomes, analyses) to relevant parties (eg, investigators, REC/IRBs, trial participants, trial registries, journals, regulators)	______4________

Consent or assent	26a	Who will obtain informed consent or assent from potential trial participants or authorised surrogates, and how (see Item 32)	_____8_______
	26b	Additional consent provisions for collection and use of participant data and biological specimens in ancillary studies, if applicable	_____n/a______
Confidentiality	27	How personal information about potential and enrolled participants will be collected, shared, and maintained in order to protect confidentiality before, during, and after the trial	______11______
Declaration of interests	28	Financial and other competing interests for principal investigators for the overall trial and each study site	______15______
Access to data	29	Statement of who will have access to the final trial dataset, and disclosure of contractual agreements that limit such access for investigators	______11_______
Ancillary and post-trial care	30	Provisions, if any, for ancillary and post-trial care, and for compensation to those who suffer harm from trial participation	______-_______
Dissemination policy	31a	Plans for investigators and sponsor to communicate trial results to participants, healthcare professionals, the public, and other relevant groups (eg, via publication, reporting in results databases, or other data sharing arrangements), including any publication restrictions	_____14_______
	31b	Authorship eligibility guidelines and any intended use of professional writers	______-______
	31c	Plans, if any, for granting public access to the full protocol, participant-level dataset, and statistical code	_____-_______
**Appendices**			
Informed consent materials	32	Model consent form and other related documentation given to participants and authorised surrogates	__on request_
Biological specimens	33	Plans for collection, laboratory evaluation, and storage of biological specimens for genetic or molecular analysis in the current trial and for future use in ancillary studies, if applicable	_____n/a______

*It is strongly recommended that this checklist be read in conjunction with the SPIRIT 2013 Explanation & Elaboration for important clarification on the items. Amendments to the protocol should be tracked and dated. The SPIRIT checklist is copyrighted by the SPIRIT Group under the Creative Commons "Attribution-NonCommercial-NoDerivs 3.0 Unported" license.
